# Integrated Metabolomics-KPCA-Machine Learning framework: a solution for geographical traceability of Chinese Jujube

**DOI:** 10.1016/j.fochx.2025.103069

**Published:** 2025-09-23

**Authors:** Xiaoli Wang, Xiaolei Ma, Yuxin Liu, Wenhan Tao, Yuting Zuo, Yueqin Zhu, Feng Hua, Chanming Liu, Wei Huang

**Affiliations:** Changzhou Municipal Hospital of Traditional Chinese Medicine, Nanjing University of Chinese Medicine, Changzhou 213003, China

**Keywords:** Chinese jujube, Metabolomics, LC-MS/MS, Kernel principal component analysis, K-means clustering, Machine learning, Traceability

## Abstract

Due to widespread product adulteration, Chinese jujube (CJ), a crop of global economic importance with nutritional and medicinal properties, struggles with geographical traceability. The study introduced a Metabolomics-Kernel Principal Component Analysis (KPCA)-Machine Learning (ML) framework to set up an origin identification system for CJ from six production regions in China (Xinjiang, Gansu, Shaanxi, Henan, Shandong, and Hebei). Using LC-MS/MS for untargeted metabolomics, researchers identified 312 metabolites. Multivariate analysis revealed 37 key discriminant variables (VIP > 1). KPCA compressed these features into 28 principal components (retaining 90.59 % information). Compared with the traditional method, the K-means clustering after dimensionality reduction of KPCA greatly improves the sample differentiation ability: the origin samples with original data overlap with fuzzy boundaries; while after dimensionality reduction, the six origin samples form a clear and compact cluster, which achieves accurate classification. This study pioneers a “Metabolomics-KPCA-ML” paradigm, offering a solution for traceability of geographical indication agricultural products.

## Highlights

• Developed a pioneering “Metabolomics-KPCA-Machine Learning” framework.
• Identified 312 metabolites, revealing region-specific metabolic fingerprints of CJ.
• KPCA-driven model greatly improves the CJ samples differentiation ability.
• This study provides a technological solution for combating geographic mislabelling.

## Graphical abstract

**Figure:**
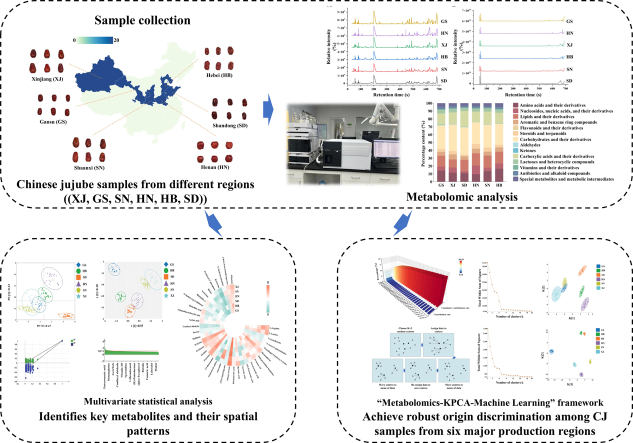

## 1 Introduction

The jujube tree (*Ziziphus jujuba* Mill.), a globally significant economic crop, has developed a vast germplasm resource pool encompassing over a thousand subspecies (Liu et al., 2015). It is found in subtropical and tropical areas throughout Asia, the Americas, and along the Mediterranean coast (Xue et al., 2022). This species, characterized by its thermophilic and hydrophilic biological traits—optimal growth occurring at temperatures between 25 and 32 °C and requiring an annual water input of 400 to 800 mm—exhibits significant environmental adaptability. It successfully completes its lifecycle across ecological gradients with mean annual temperatures ranging from 8 to 24 °C and annual precipitation levels between 60 and 1600 mm (Luo et al., 2023; Song et al., 2019). The Chinese jujube (CJ), a distinctive medicinal and edible species within the Rhamnaceae family, is categorized alongside chestnut, peach, apricot, and plum as one of the “Five Fruits” (Hou et al., 2022). The fruits contain cyclic nucleotides, triterpenoids, and various vitamins, creating a distinctive nutritional and pharmacological value system (Ji et al., 2020; Muhammad et al., 2024). The global jujube industry demonstrates a “unipolar diversity” development pattern. Despite the fact that cultivation rights have been extended to over 50 countries, China predominates in international markets, boasting an annual production of 7.5 million tons, which accounts for over 90 % of the global output. Additionally, China benefits from extensive advantages across the entire industrial chain (Cai et al., 2024). Its primary production areas are concentrated in the temperate monsoon climate zones of northern China (Huang et al., 2015).

As the principal cultivated subspecies, the CJ has developed into an ecological population along the Yellow River over four millennia of artificial selection (Wu et al., 2023). Highly esteemed as both a culinary delicacy and a component of traditional Chinese medicine, CJ is renowned for its remarkable nutritional and medicinal attributes. Recent studies have demonstrated its extensive range of biological activities, encompassing anti-cancer, anti-epileptic, anti-inflammatory, and neuroprotective effects, as well as its potential to alleviate insomnia (Lu et al., 2021). CJ cultivation in northern China demonstrates a “dual-core driven” industrial framework. The traditional eastern production areas, including Henan, Hebei, Shandong, and Shaanxi, utilize fertile alluvial plains to support intensive agricultural practices. In contrast, the emerging western regions, such as Xinjiang and Gansu, are pioneering water-saving cultivation models that capitalize on the light and heat resources characteristic of arid zones (Lu et al., 2023; Wu et al., 2023). CJ is commonly marketed as fresh or dried fruit, with drying being the predominant processing method (Yang et al., 2021). Notably, sensory quality, nutritional composition, and flavor profiles vary significantly among ecological types of CJ (Lu et al., 2021). Increasing public awareness of health issues has intensified concerns regarding food traceability, primarily due to the prevalent geographic mislabeling and adulteration found in commercial CJ products (Walaszczyk et al., 2022).

Metabolomics, a pivotal branch of systems biology, has opened new dimensions for agricultural product traceability (Alseekh et al., 2018; Tian et al., 2020). The systematic analysis of low-molecular-weight metabolites (<1000 Da) within biological systems has emerged as a pivotal technology for elucidating plant physiological states and varietal characteristics. Owing to its high sensitivity and comprehensive analytical capabilities, this approach offers distinct advantages in the identification of fruit germplasm, the metabolic profiling of developmental stages, and the discovery of biomarkers (Chen et al., 2023; Tang et al., 2020; Xu et al., 2021). In comparison to genomics and transcriptomics, metabolites, as the terminal products of biological systems, provide a direct reflection of phenotypic characteristics. Their dynamic alterations offer precise insights into the physiological states of fruits and the mechanisms underlying environmental responses. Nevertheless, the extensive high-dimensional data produced by metabolomic studies, which typically encompass hundreds to thousands of metabolic features, present substantial challenges to conventional analytical methods, especially in tracing the geographical indications of complex products (Martínez-Rivas & Fernie, 2024). Machine learning (ML) technologies provide revolutionary solutions for metabolomic data complexity (Gong et al., 2023). Utilizing feature selection and pattern recognition algorithms, ML proficiently mitigates environmental noise, identifies essential metabolite variables, and interprets nonlinear interactions within metabolic networks. Research indicates that ensemble learning algorithms, such as Random Forest (RF), address biological sample variability through multi-decision tree modeling, thereby facilitating a robust ranking of feature importance (Cai et al., 2023). Kernel-based methods for nonlinear dimensionality reduction, including Kernel Principal Component Analysis (KPCA), outperform traditional Principal Component Analysis (PCA) in managing complex metabolic correlations. By projecting raw data into high-dimensional feature spaces, KPCA more effectively captures nonlinear patterns among metabolites (Shiokawa et al., 2018). These technological innovations introduce new analytical dimensions to food multi-omics research, facilitating significant advancements in the traceability of agricultural products, the prediction of flavor profiles, and the monitoring of storage quality.

This study examines commercial CJ samples from key production regions in China, namely Xinjiang, Gansu, Shaanxi, Henan, Shandong, and Hebei. It introduces an innovative analytical framework that integrates metabolomics, KPCA and ML. By utilizing KPCA for nonlinear dimensionality reduction, the original metabolic feature space is condensed into a core principal component space. This enables the creation of a metabolic network-based traceability decision system capable of multi-level geographical discrimination at the provincial, county, and plantation levels. This multidimensional, multi-algorithm approach offers a novel technical pathway for food traceability research, with the potential to achieve significant advancements in the tracking of jujube origins and quality authentication. Additionally, it provides robust technical support for the protection of geographical indication products.

## 2 Materials and methods

### 2.1 Sample collection

**Fig. 1:**
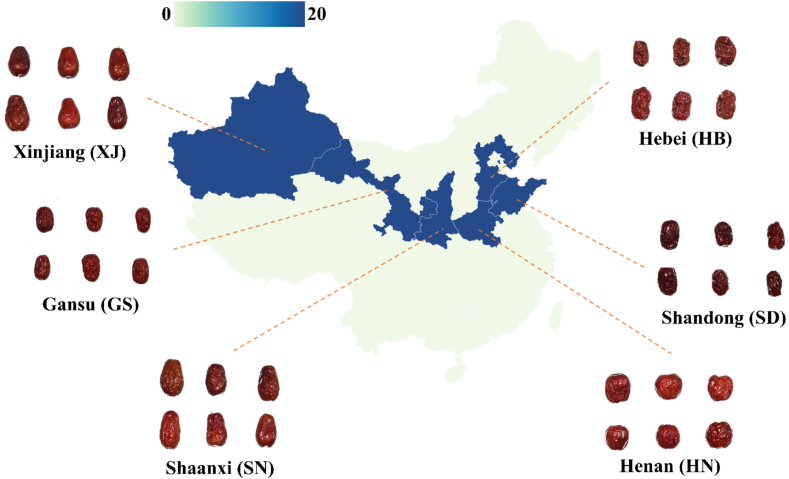
Geographic information of CJ samples.

GS, Gansu; HB, Hebei; SD, Shandong; SN, Shaanxi; HN, Henan; XJ, Xinjiang (the same as below).

From six major production regions in northern China, namely Xinjiang (XJ), Gansu (GS), Shaanxi (SN), Henan (HN), Hebei (HB), and Shandong (SD), a total of 120 mature CJ fruit samples were collected, with 20 batches per region. After surface impurity removal via deionized water rinsing, samples were dried at 45 °C for 1 h to eliminate residual moisture and stored at −20 °C to prevent metabolite degradation. All samples carried unique geographical identifiers (XJ-CJ, GS-CJ, SN-CJ, HN-CJ, HB-CJ, or SD-CJ), with coordinates spanning 33.63°N–41.17°N latitude and 79.93°E–117.97°E longitude (Table S1 and Fig. 1). Reagents included methanol (Thermo, ≥99.0 %, CAS 67–56-1), 2-chloro-*L*-phenylalanine (Aladdin, 98 %, CAS 103616–89-3), acetonitrile (Thermo, ≥99.9 %, CAS 75–05-8), formic acid (TCI, LC-MS grade, CAS 64–18-6), and ammonium formate (Sigma, ≥99.9 %, CAS 540–69-2).

### 2.2 Sample preparation and metabolite extraction

After de-pitting and vacuum freeze-drying, CJ samples were pulverized using a Retsch MM400 grinder (Germany). A precise amount of 50 mg of powder was placed into 2 mL centrifuge tubes, mixed with 600 μL of methanol that included 4 ppm 2-chloro-*L*-phenylalanine as an internal standard, and vortexed for 30 s. Following cell disruption with 3 mm steel beads in a tissue homogenizer (55 Hz, 60 s), samples underwent 15 min room-temperature ultrasonic extraction. After centrifugation (12,000 rpm, 10 min, 4 °C), supernatants were filtered through 0.22 μm nylon membranes into injection vials, with three biological replicates per sample.

### 2.3 Instrumental analysis

#### 2.3.1 Chromatographic conditions

Equipped with an ACQUITY UPLC® HSS T3 column (2.1 × 100 mm, 1.8 μm; Waters, USA), the Thermo Vanquish UPLC system from Thermo Fisher Scientific, USA, operated at a flow rate of 0.3 mL/min and a temperature of 40 °C. An injection volume of 2 μL was used. For positive ion mode, the mobile phases included 0.1 % formic acid in acetonitrile (B2) and 0.1 % formic acid in water (A2), with a gradient elution: 0–1 min at 8 % B2; 1–8 min from 8 % to 98 % B2; 8–10 min at 98 % B2; 10–10.1 min, 98 %–8 % B2; 10.1–12 min, 8 % B2. In negative ion mode, acetonitrile (B3) and 5 mM ammonium formate (A3) were utilized, following the same gradient programming.

#### 2.3.2 Mass spectrometric conditions

The Thermo Orbitrap Exploris 120 mass spectrometer, manufactured by Thermo Fisher Scientific in the USA, alternated between positive (3.50 kV spray voltage) and negative (−2.50 kV) ion modes with an ESI source. The settings include a sheath gas of 40 arb, auxiliary gas of 10 arb, and a capillary temperature of 325 °C. Full-scan MS1 (*m*/*z* 100–1000) was performed at 60,000 resolution, with HCD fragmentation (30 % collision energy) and MS2 acquisition (15,000 resolution) of the top four ions, employing dynamic exclusion to minimize redundant MS/MS data.

### 2.4 Metabolite identification and quantification

Using Proteowizard MSConvert (v3.0.8789), raw MS files were converted to mzXML format. XCMS (v3.12.0) in R handled peak detection, filtering, and alignment with parameters including bw = 2, ppm = 15, peakwidth = c(5,30), mzwid = 0.015, mzdiff = 0.01, and method = ‘centWave’. Total peak area normalization corrected systematic variations. Metabolite identification utilized HMDB, MassBank, LipidMaps, mzCloud, and KEGG databases (mass error < 30 ppm). Peak integration/calibration used MultiQuant software, with chromatographic peak areas representing relative metabolite abundances.

### 2.5 KPCA dimensionality reduction

This study employs KPCA to address the complexity of nonlinear distributions in high-dimensional datasets by reducing the dimensionality of the original features. KPCA improves traditional PCA by using kernel methods to project data into a high-dimensional RKHS, enabling linear projections to effectively capture nonlinear structures. Unlike PCA, which only identifies linear correlations, KPCA uses nonlinear kernel functions like Gaussian and polynomial kernels to detect complex patterns (Shi, Han, et al., 2022). This capability reduces redundancy and retains key nonlinear features for classification, mitigating the “curse of dimensionality” and offering a compact, distinct input for future models. As an essential preprocessing step, the low-dimensional features generated by KPCA will be utilized in the classification models discussed in ‘Section 2.6’, to enhance classification performance. This framework of “nonlinear dimensionality reduction combined with discriminative models” seeks to balance feature expressiveness with model complexity, thereby achieving improved accuracy and efficiency in high-dimensional classification tasks. The detailed operational procedures and key steps of the KPCA dimension reduction algorithm used in this study are listed in Table S2.

### 2.6 Discriminative models

In this study, the K-means clustering algorithm, grounded in the unsupervised learning paradigm, has been selected for analysis. Recognized as a quintessential method within contemporary data science and machine learning, this algorithm is employed to categorize a complex multidimensional dataset into K distinct and relatively homogeneous clusters. This is achieved through an iterative optimization strategy (Chen et al., 2024). The mathematical model underlying the algorithm is designed to attain the optimal partitioning of data points. The central computational mechanism can be delineated through the following key steps: initially, K preliminary cluster centers are randomly chosen to serve as the starting point for the iteration. Subsequently, an iterative process ensues, wherein data points are assigned to clusters and cluster centers are recalculated, with the objective of progressively converging to a stable configuration of the clusters (Chen et al., 2020). In each iteration, the algorithm precisely computes the distances between data points and cluster centers, subsequently assigning the data points to the nearest clusters. It then recalculates the geometric centers of these clusters, thereby establishing a dynamic and adaptive clustering evolution process. The theoretical foundation of the K-means algorithm is rooted in minimizing the sum of squared distances from intra-cluster points to their respective cluster centers. This aims to create clusters that exhibit high internal homogeneity while maintaining significant differences between distinct clusters. Nonetheless, this method is not devoid of limitations (Sheng et al., 2022). The algorithm necessitates that the researcher pre-determine the number of clusters, K, a requirement that frequently presents a methodological challenge in practice. Additionally, the algorithm is notably sensitive to the selection of initial centroids, with varying initialization configurations potentially resulting in substantially different clustering outcomes. To mitigate this intrinsic limitation, multiple random initialization strategies are commonly employed, and quantitative techniques such as the elbow method and silhouette coefficients are utilized to rigorously evaluate cluster number selection and clustering quality. Despite these challenges, K-means retains significant analytical value across various fields, particularly in the context of large-scale, high-dimensional data scenarios, where its computational efficiency and algorithmic simplicity are especially advantageous. In contemporary machine learning practice, K-means is often integrated with dimensionality reduction techniques, such as Principal Component Analysis, to enhance the accuracy and depth of data analysis insights (Chen et al., 2024).

### 2.7 Data analysis

The data are expressed as mean ± standard deviation (SD). Statistical analyses were performed using SPSS software (Version 22, SPSS Inc.). Analysis of variance (ANOVA) was employed alongside Duncan's multiple range test and the least significant difference test to identify statistically significant differences (*P* < 0.05). Principal Component Analysis (PCA) was conducted using Simca software (Version 14.1, Sartorius Group, SW), while partial least squares discriminant analysis (PLSDA) was executed with GraphPad Prism (Version 9, GraphPad Software, San Diego, CA, USA). Furthermore, classification algorithm analyses utilizing Kernel Principal Component Analysis (KPCA) and K-means clustering were carried out using MATLAB software (R2023a, The MathWorks, Inc., USA).

## 3 Results and discussion

### 3.1 Applicability of LC-MS/MS in CJ metabolite analysis

**Fig. 2:**
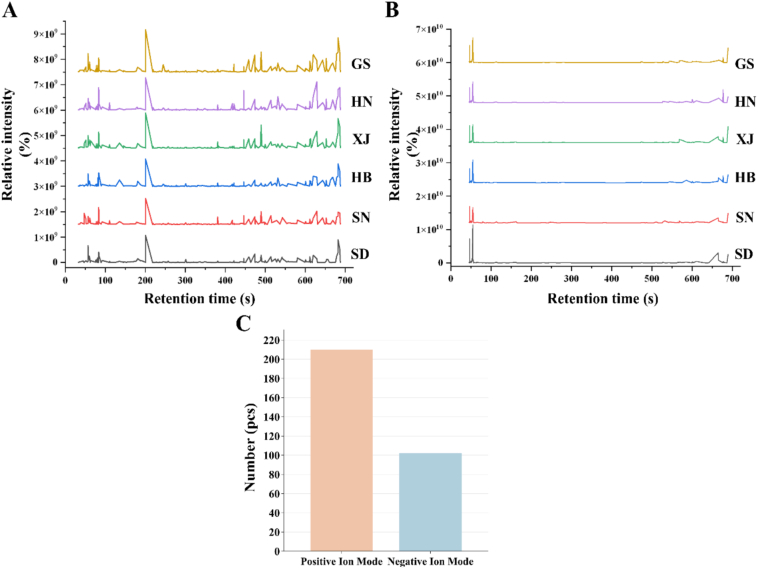
Positive ion mode total ion flow chromatogram (A); Negative ion mode total ion flow chromatogram (B); Metabolite identification of CJ samples based on LC-MS/MS untargeted metabolomics (positive/negative ion mode distributions) (C).

Ultra-performance liquid chromatography-tandem mass spectrometry (LC-MS/MS) was utilized to conduct a systematic analysis of non-volatile metabolites in jujube samples sourced from six distinct production regions. As depicted in Fig. 2, the total ion chromatograms (TIC) in both positive ion mode (Fig. 2-A) and negative ion mode (Fig. 2-B) exhibited highly similar profiles across the different regions, with the main peak retention times and shapes demonstrating significant consistency (relative standard deviation, RSD <5 %). This observation indicates that the core metabolite compositions are largely conserved, likely governed by metabolic pathways essential during fruit development, such as glycolysis, the tricarboxylic acid (TCA) cycle, and phenylpropanoid biosynthesis. Nevertheless, subtle variations in local peak intensities and retention times suggest potential differences in the abundance or types of specific metabolites among geographically distinct samples. Employing a non-targeted metabolomics approach combined with database matching (HMDB, MassBank, LipidMaps, mzCloud, and KEGG), a total of 312 metabolites were identified, with 210 detected in positive ion mode and 102 in negative ion mode (Fig. 2-C). The mass spectra match, retention times, and mass errors for specific metabolites are comprehensively detailed in Table S3. These findings suggest that the non-volatile metabolite profiles of CJ samples from northern China demonstrate substantial regional commonality. However, the nuanced variations in trace components may serve as essential chemical markers for geographical traceability.

### 3.2 Composition analysis of non-volatile metabolites in CJ based on LC-MS/MS

**Fig. 3:**
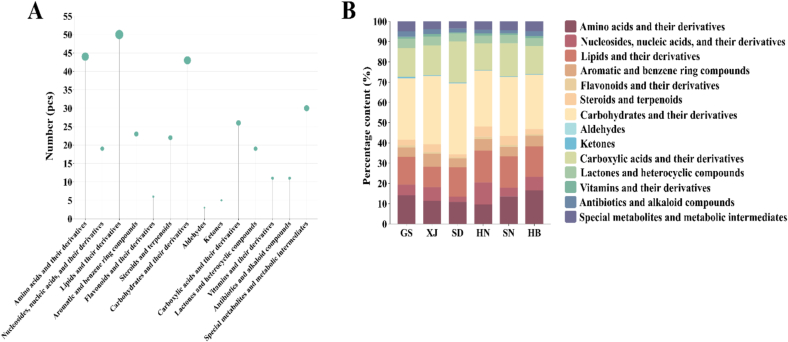
Functional classification and distribution characteristics of metabolites CJ samples (A), and stacked histogram of metabolic composition of CJ samples (B).

The 312 identified metabolites were systematically categorized into 14 functional groups based on their chemical structure, biosynthetic pathways, biological functions, and metabolic stages, as illustrated in Fig. 3-A. These groups include: amino acids and their derivatives (44), nucleosides, nucleic acids, and their derivatives (19), lipids and their derivatives (50), aromatic and benzene ring compounds (23), flavonoids and their derivatives (6), sterols and terpenoids (22), carbohydrates and their derivatives (43), aldehydes (3), ketones (5), carboxylic acids and their derivatives (26), lactones and heterocyclic compounds (19), vitamins and their derivatives (11), antibiotics and alkaloid compounds (11), and special metabolites and metabolic intermediates (30). The analysis indicated that lipids and their derivatives constituted the most abundant category, whereas aldehydes were the least represented.

Stacked bar charts illustrating the relative abundances of the 14 metabolite categories (Fig. 3-B) revealed significant compositional similarities among CJ samples from all six regions. The cumulative relative abundances of amino acids and their derivatives, lipids and their derivatives, carbohydrates and their derivatives, and carboxylic acids and their derivatives surpassed 65.00 % across all regions. The metabolic composition of CJ represents a complex biochemical system, where amino acids, as fundamental units of proteins, not only determine the nutritional value of CJ but also play a crucial role in physiological activities such as antioxidant processes and plant growth regulation (Guo et al., 2015). Lipid derivatives affect the flavor and nutritional value, and unsaturated fatty acids can enhance the quality of products (Gao et al., 2012). Sugar derivatives are critical constituents of Chinese jujube (CJ), with fructose and glucose serving as the primary components. These sugars not only impart the distinctive sweetness characteristic of jujube but also act as an energy source and enhance its flavor profile (Shi et al., 2018). Research indicates significant variability in the sugar content of jujube from different geographical origins, with relative content ranging from 26.76 % to 35.07 %. Notably, samples from Shandong (SD) and Xinjiang (XJ) origins exhibit pronounced sugar enrichment. This elevated sugar content not only augments the flavor quality of jujube but may also contribute to its antioxidant properties. Despite the relatively low presence of carboxylic acid derivatives in jujube, their functional roles should not be underestimated (Guo et al., 2015). The organic acids, specifically malic acid and citric acid, play a crucial role in modulating the acidity and flavor profile of jujube. Additionally, they are intimately associated with the fruit's storage stability and processing attributes. Collectively, these acids contribute to the distinct nutritional value and quality characteristics of CJ (Xue et al., 2021). The synergistic interaction of these components enhances the nutritional richness and unique flavor of jujube, offering essential data support for the development of a metabolite-based origin discrimination system.

The analysis of specific regional metabolic profiles revealed distinct compositions. In Gansu (GS), the predominant metabolites were carbohydrates and their derivatives, constituting 30.37 % of the profile, followed by carboxylic acids and their derivatives (14.15 %), amino acids and their derivatives (14.11 %), and lipids and their derivatives (13.78 %). In contrast, Hebei (HB) exhibited a metabolic composition primarily consisting of carbohydrates and their derivatives at 26.76 %, with amino acids and their derivatives at 16.52 %, lipids and their derivatives at 15.03 %, and carboxylic acids and their derivatives at 13.84 %. Shandong (SD) demonstrated the highest concentration of carbohydrates and their derivatives at 35.07 %, followed by carboxylic acids and their derivatives at 20.21 %, lipids and their derivatives at 14.41 %, and amino acids and their derivatives at 10.77 %. In Henan (HN), carbohydrates and their derivatives were the most prevalent category, accounting for 27.58 % of the composition. This was followed by lipids and their derivatives at 15.61 %, carboxylic acids and their derivatives at 13.20 %, and amino acids and their derivatives at 9.58 %, in descending order. In Shaanxi (SN) and Xinjiang (XJ), carbohydrates and their derivatives also predominated, constituting 29.18 % and 33.63 %, respectively. In SN, the secondary constituents included carboxylic acids and their derivatives (16.36 %), lipids and their derivatives (15.38 %), and amino acids and their derivatives (13.57 %). Conversely, in XJ, a more distinct gradient was observed, with carboxylic acids and their derivatives at 14.64 %, lipids and their derivatives at 11.40 %, and amino acids and their derivatives at 10.11 %.

A detailed analysis uncovered notable spatial heterogeneity in the distribution of region-specific metabolites. For instance, Henan (HN) exhibited elevated concentrations of secondary metabolites, including nucleosides, nucleic acids, and their derivatives (10.93 %), flavonoids and their derivatives (1.20 %), and steroids and terpenoids (5.08 %). In contrast, Shandong (SD) presented the lowest levels of these compounds, with percentages of 2.68 %, 0.48 %, and 1.50 %, respectively. Xinjiang (XJ) showed the highest concentrations of aromatic and benzene ring compounds (6.39 %) and vitamins and their derivatives (1.26 %), while recording the lowest aldehyde content (0.04 %). Gansu (GS) was distinguished by its elevated levels of defense-related metabolites, such as ketones (0.70 %), lactones and heterocyclic compounds (4.67 %), and antibiotics and alkaloid compounds (2.72 %), as well as higher concentrations of specialized metabolites and metabolic intermediates (4.88 %). Shandong (SD) consistently exhibited the lowest levels across most secondary metabolite categories, possibly due to ecological factors influencing metabolic pathways. These spatially varied metabolic profiles provide crucial chemical fingerprinting evidence for tracing the origin of CJ (Shi, Shen, et al., 2022).

### 3.3 Multivariate statistical analysis

**Fig. 4:**
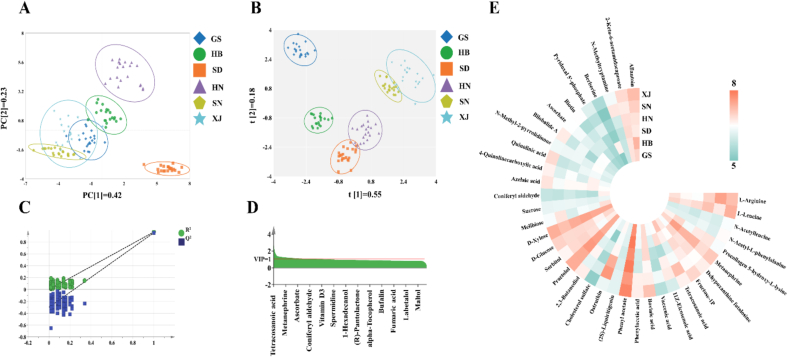
PCA model score plots (A); PLS-DA model score plots (B); permutation test cross-validation results with 200 calculations (C); VIP plots (D); heat map of differential metabolites in CJ samples (E).

A comprehensive metabolite database was developed and subjected to multivariate statistical analysis to ensure the reliability of traceability. This study utilized multivariate statistical methods to clarify subtle differences in the intrinsic structure of metabolite data from CJ samples collected from six regions: Xinjiang, Gansu, Shaanxi, Henan, Hebei, and Shandong. The findings are presented in Fig. 4. In recent years, PCA has emerged as a powerful method for data dimensionality reduction and has been extensively utilized in research focused on the identification of varieties and geographical origins of agricultural products (Abdelhafez et al., 2020). In the PCA depicted in Fig. 4-A, the first and second principal components accounted for 42 % and 23 % of the total variance, respectively, culminating in a cumulative contribution of 65 %. The analysis revealed distinct clustering patterns among the six origin samples: the SD and HN samples formed the most distinct clusters, whereas the HB, GS, XJ, and SN samples exhibited some degree of overlap in their spatial distribution. This complex distribution pattern suggests both significant intrinsic feature differences and potential similarities among samples from different origins, which may be attributed to analogous geological and climatic conditions across regions.

In contrast to conventional unsupervised PCA techniques, partial least squares discriminant analysis (PLS-DA), as a supervised learning approach, offers enhanced accuracy in disentangling overlapping information and identifying the key metrics that significantly contribute to the principal components through attribution analysis (Jiménez-Carvelo et al., 2021). As depicted in Fig. 4-B, the first discriminant component, t [1], accounts for 55 % of the discriminant variance, while the second discriminant component, t [2], accounts for 18 %, resulting in a cumulative discriminant rate of 73 %. In comparison to PCA, PLS-DA markedly enhanced sample clustering by reducing intra-sample dispersion and increasing inter-group separation. However, some overlap persists between the SD and HN samples, as well as between the SN and XJ samples, indicating that complete separation was not achieved. To mitigate the risk of overfitting during the modeling process, a permutation test was conducted to evaluate the efficacy of the supervised model. Depicted in Fig. 4-C are the permutation test results for the PLS—DA model within the comparison group. The noted decline in permutation retention, coupled with decreases in the R^2^ and Q^2^ values of the stochastic model, suggests that the model is robust and not subject to overfitting (Birenboim et al., 2023).

The analysis identified 37 metabolites as discriminatory, characterized by VIP scores exceeding 1 and significance levels below 0.05 (Xu et al., 2024) (Fig. 4-D). These metabolites include L-Arginine, L-Leucine, N-Acetylleucine, N-Acetyl-*L*-phenylalanine, Procollagen 5-hydroxy-l-lysine, Metanephrine, Dehypoxanthine futalosine, Fructose-1P, Tetracosanoic acid, 11Z-Eicosenoic acid, Vaccenic acid, Bovinic acid, Phenylacetic acid, Phenyl acetate, (2*S*)-Liquiritigenin, Ostruthin, Cholesterol sulfate, 2,3-Butanediol, Practolol, Sorbitol, d-Glucose, D-Xylose, Melibiose, Sucrose, Coniferyl aldehyde, Azelaic acid, 4-Quinolinecarboxylic acid, Quinolinic acid, N-Methyl-2-pyrrolidinone, Bilobalide A, Ascorbate, Biotin, Pyridoxal 5′-phosphate, Berberine, N-Methyltryptamine, 2-Keto-6-acetamidocaproate, and Allantoin.

The heatmap analysis presented in Fig. 4-E demonstrated distinct region-specific expression patterns. CJ samples from various regions (SN, XJ, HB, GS, HN, SD) develop distinct metabolic profiles and region-specific chemical “fingerprints” due to variations in environmental factors like climate, soil, water, light, and biotic stress. CJ samples from arid, sunny regions (XJ and GS) are high in flavonoids and aromatic compounds like (2*S*)-Liquiritigenin and Phenylacetic acid, which help resist UV radiation and add a distinct aroma (Lu et al., 2023). In XJ, the significant temperature variation and ample sunlight lead jujubes to accumulate sugars (d-Glucose, Sucrose), boosting sweetness and texture (Shi et al., 2018). CJ samples from humid and hot regions (HN) contain compounds like Practolol, Sorbitol, and Pyridoxal 5′-phosphate, which help with osmotic regulation and pest/disease defense in wet, disease-prone environments. CJ samples from the dry, sunny SN region accumulate L-Arginine, Tetracosanoic acid, Ostruthin, and N-Methyltryptamine, providing drought resistance, UV protection, and chemical defense, along with nutritional and pharmacological benefits (Ji et al., 2020; Muhammad et al., 2024). In temperate humid regions (HB), CJ samples are rich in diverse compounds like amino acid derivatives, long-chain fatty acids, aromatic compounds, defensive alkaloids, and antioxidants, indicating strong chemical defense and rich flavor (Lu et al., 2021). CJ samples from coastal temperate regions (SD) are marked by D-Xylose, suggesting a metabolic focus on monosaccharides, likely due to the local mild climate and optimal light conditions. These environmentally influenced metabolic variations help trace the origin and quality of jujubes from various regions, supporting geographical indication protection and the creation of unique brands.

### 3.4 KPCA dimensionality reduction analysis

While multivariate statistical analysis has proven to be of significant value in metabolomics research, its practical application is hindered by two primary technical challenges. First, traditional linear models exhibit limited capacity to address the nonlinear characteristics inherent in complex metabolic networks, thereby constraining the accuracy of origin discrimination, which in this study is recorded at 82.3 %. Second, the high dimensionality of metabolic data, encompassing 312-dimensional features in this study, substantially increases computational complexity and adversely impacts analytical efficiency. To effectively address these challenges, this study introduces an innovative approach by employing the KPCA dimensionality reduction algorithm, with the objective of enhancing both the accuracy and efficiency of data processing (Liu et al., 2019).

**Fig. 5:**
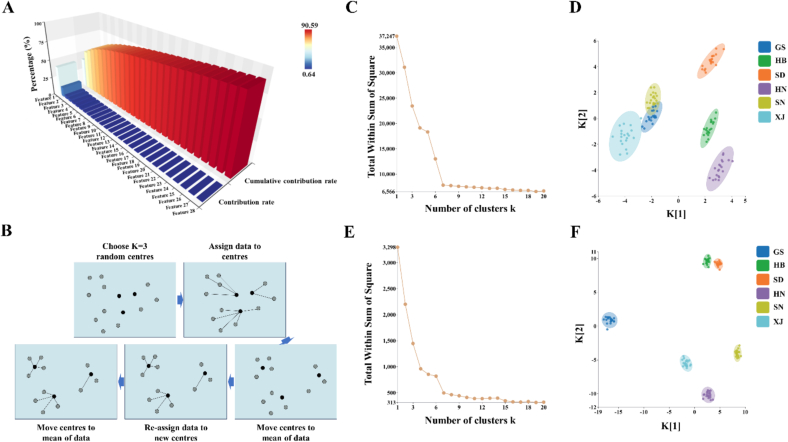
Histogram of the contribution and Cumulative contribution of KPCA downscaled feature data in CJ samples(A); K-means Algorithm Illustration / Mechanism of K-means Clustering (B); Elbow Plot for K-means on Original High-Dimensional Data (C); K-means Clustering Results on Original High-Dimensional Data (k = 6) (D); Elbow Plot for K-means on KPCA-Reduced Feature Data (E); K-means Clustering Results on KPCA-Reduced Feature Data (k = 6) (F).

KPCA represents a sophisticated nonlinear dimensionality reduction method that utilizes a kernel function to map original low-dimensional data, which is not linearly separable, into a high-dimensional feature space, thereby enabling the nonlinear reduction of complex datasets (Wang et al., 2021). In comparison to traditional PCA, KPCA offers notable advantages: it effectively captures the nonlinear characteristics of data, substantially reduces dimensionality while preserving essential information, and demonstrates greater adaptability to high-dimensional, non-Gaussian distributed data. In this study, the KPCA analysis results (Fig. 5-A) indicate that the original metabolic feature space, derived from a 312-dimensional metabolite database encompassing 120 CJ sample batches from six different origins, can be optimized into 28 core principal components when the cumulative contribution threshold is set to 90 %. The 28 principal components effectively preserved 90.59 % of the informative content inherent in the original dataset, achieving a feature dimensionality reduction rate of 91.03 %. By employing a kernel function, specifically the Radial Basis Function (RBF) kernel with a parameter γ = 0.05, the method nonlinearly transforms the original 312-dimensional metabolic data into a 28-dimensional feature space. This approach facilitates efficient data simplification while maximizing information retention.

### 3.5 Machine learning classification algorithm used for backward validation analysis

Utilizing the 28-dimensional feature matrix derived through dimensionality reduction, this study further assessed the efficacy of Kernel Principal Component Analysis (KPCA) in elucidating sample structure and determining origin specificity. To achieve this, unsupervised K-means clustering analysis was conducted on 120 batches of CJ samples originating from six distinct geographical regions (GS, HB, SD, HN, SN, XJ). This analysis employed both the original 312-dimensional metabolomic data and the reduced-dimensional features extracted via KPCA. Fig. 5-B effectively delineates the fundamental operational mechanism of the K-means clustering algorithm. Initially, a predetermined number of initial cluster centers (K = 3 in this illustration) are selected at random (Duan et al., 2022). The algorithm then iteratively executes two principal steps: first, each data point is allocated to the nearest cluster center; second, the position of each cluster center is recalibrated to the centroid of the data points assigned to it. This iterative process of assignment and recalibration continues until the cluster centers stabilize, with the objective of partitioning the dataset into K distinct and cohesive clusters (Chen et al., 2020; Chen et al., 2024).

The direct application of K-means clustering to unprocessed high-dimensional metabolomic data is suboptimal. As illustrated in Fig. 5-C, the elbow plot, despite displaying an indistinct ‘elbow’ near (k = 6), which implies that six clusters might be appropriate, initially exhibits a significantly high total intra-cluster sum of squares (WSS). This observation suggests a considerable degree of complexity or dispersion within the internal structure of the data. The clustering results presented in Fig. 5-D substantiate this observation: although attempts were made to categorize the samples into six distinct groups, there is substantial overlap among the sample points from GS, XJ, and SN, rendering them challenging to distinguish. Conversely, samples from other regions, such as Hebei (HB), Shandong (SD), and Henan (HN), exhibit the formation of approximate clusters; however, these clusters display considerable dispersion and indistinct boundaries. This suggests that the high dimensionality and noise inherent in the original dataset impeded the K-means algorithm's capacity to accurately discern natural groupings based on origin. In contrast, K-means cluster analysis utilizing the KPCA-reduced feature matrix reveals marked improvements in performance. The elbow plot presented in Fig. 5-E indicates that the data processed through Kernel Principal Component Analysis (KPCA) exhibits a lower initial Within-Cluster Sum of Squares (WSS) and reveals a distinctly pronounced ‘elbow’ at k = 6. This observation strongly endorses the partitioning of the data into six clusters. Furthermore, the clustering outcomes depicted in Fig. 5-F illustrate an almost perfect separation: samples from all six geographical origins (GS, HB, SD, HN, SN, XJ) form distinct, internally cohesive, and well-defined clusters, with the previously existing overlap being entirely eliminated. This outcome demonstrates that KPCA has effectively extracted the critical low-dimensional features pertinent to the origin, significantly optimizing the data structure. Consequently, the K-means algorithm is able to accurately classify the samples into groups based on their geographic origins, as inferred from the metabolite profiles.

Furthermore, this study conducted an analysis utilizing both the original high-dimensional data and the dimensionally-reduced data, processed via kernel principal component analysis (KPCA), as inputs. These inputs were employed in conjunction with several prominent machine learning classification algorithms, including support vector machines (SVM), deep belief networks (DBN), and random forests (RF). The pertinent results are depicted in Fig. S1. Validation on the same dataset facilitated a comparative assessment of the classification performance across different algorithms, revealing that K-means surpassed other methods in multiple evaluation metrics, such as classification accuracy and precision. In contrast to various supervised learning models, K-means demonstrated greater suitability for the task of identifying the origin of CJ samples from six major production regions in this investigation. It is noteworthy that K-means is an unsupervised learning technique, whereas SVM, DBN, and RF are supervised learning techniques. The findings of this study suggest that unsupervised methods, even in the absence of labeled data, can achieve superior clustering and classification performance when integrated with appropriate dimension reduction and feature extraction strategies.

## 4 Conclusions

This study established a precise system for determining the origin of CJ by integrating untargeted metabolomics, KPCA, and machine learning methodologies. Utilizing LC-MS/MS, the research systematically identified 312 metabolites from six major production regions, encompassing 14 functional categories such as amino acids, lipids, and carbohydrates, thereby highlighting significant spatial heterogeneity in the metabolic profiles of CJ across different geographical origins. Through multivariate statistical analysis, 37 discriminant biomarkers, including Tetracosanoic acid and Berberine, were identified, whose expression patterns constituted unique chemical fingerprints, offering essential evidence for origin authentication. The primary contribution of this study is the use of KPCA and the K-means clustering algorithm, which effectively addresses the nonlinear challenges of high-dimensional metabolic data. Utilizing KPCA, the initial 312-dimensional feature set was effectively reduced to 28 principal components, preserving 90.59 % of the original information while substantially optimizing the data structure. In comparison to traditional analytical methods, K-means clustering applied to the KPCA-reduced feature matrix markedly enhances the ability to differentiate samples. In the original data clustering, there is considerable overlap among samples from different regions, particularly Gansu, Xinjiang, and Shaanxi. However, following KPCA processing, the samples from the six regions form distinct clusters with well-defined and compact boundaries, thereby achieving precise differentiation. This research offers a “metabolomics-nonlinear dimensionality reduction-machine learning” framework for authenticating geographical indication products and lays the groundwork for jujube quality assessment and industry standardization.

## CRediT authorship contribution statement

**Xiaoli Wang:** Writing – original draft, Software, Methodology, Conceptualization. **Xiaolei Ma:** Writing – original draft, Software, Methodology, Conceptualization. **Yuxin Liu:** Visualization, Methodology, Investigation, Formal analysis. **Wenhan Tao:** Software, Methodology, Data curation. **Yuting Zuo:** Software, Methodology, Data curation. **Yueqin Zhu:** Software, Methodology, Data curation. **Feng Hua:** Funding acquisition, Data curation. **Chanming Liu:** Funding acquisition, Data curation. **Wei Huang:** Writing – review & editing, Software, Funding acquisition.

## Declaration of competing interest

The authors declare that they have no known competing financial interests or personal relationships that could have appeared to influence the work reported in this paper.

## Data Availability

Data will be made available on request.

## Appendix A Supplementary data

Supplementary material Image 1
