# Supplementary material for: Integrated Metabolomics-KPCA-Machine Learning framework: a solution for geographical traceability of Chinese Jujube
Source: Food Chem X. 2025 Sep 23;31:103069. doi: 10.1016/j.fochx.2025.103069 (PMC12509133; doi:10.1016/j.fochx.2025.103069)
Supplement: Supplementary file 1 — Supplementary material [file mmc1.docx]

**Supplementary materials**

**
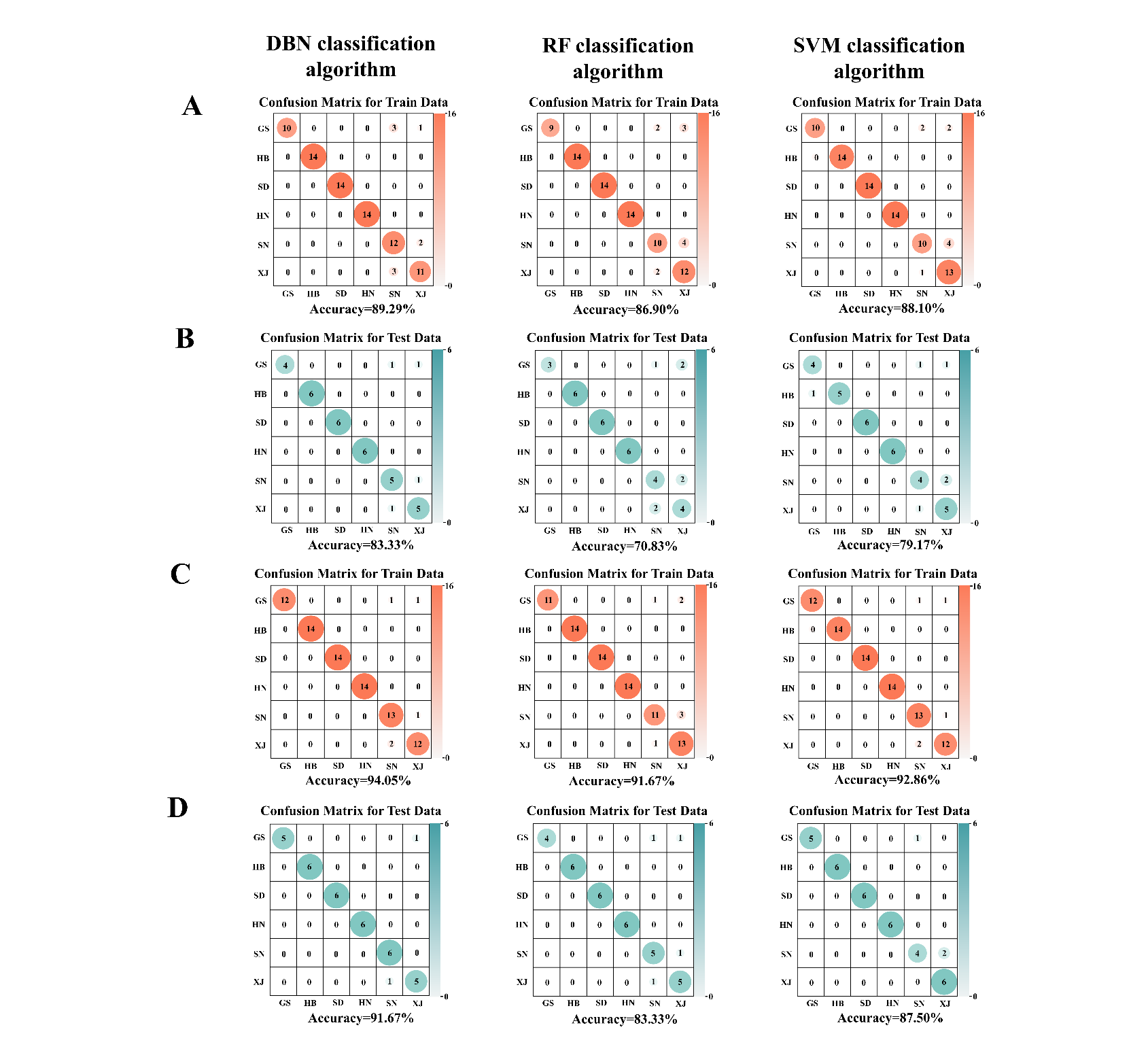
**

Figure S1. Confusion matrices of the training set (A) and test set (B) of 3 machine learning algorithms based on the original high-dimensional data for classification discrimination; Confusion matrices of the training set (C) and test set (D) of 3 machine learning algorithms based on the KPCA dimensionality reduction data for classification discrimination.

GS, Gansu; HB, Hebei; SD, Shandong; SN, Shaanxi; HN, Henan; XJ, Xinjiang.

SVM, Support Vector Machine; DBN, Deep Belief Network; RF, Random Forest.

**Table. S1** Geographic region information for Chinese jujube.

| NO. | Abbreviations | Province | City | Longitude  (˚E) | Latitude  (˚N) | Altitude  (m) | Batch number |
| --- | --- | --- | --- | --- | --- | --- | --- |
| 1 | GS | Gansu | Linze | 100.16 | 39.15 | 1312 | 202301031 |
| 2 | GS | Gansu | Linze | 100.16 | 39.15 | 1312 | 202301032 |
| 3 | GS | Gansu | Linze | 100.16 | 39.15 | 1312 | 202301033 |
| 4 | GS | Gansu | Linze | 100.16 | 39.15 | 1312 | 202301034 |
| 5 | GS | Gansu | Linze | 100.16 | 39.15 | 1312 | 202301035 |
| 6 | GS | Gansu | Zhangye | 100.45 | 38.93 | 1431 | 202302171 |
| 7 | GS | Gansu | Zhangye | 100.45 | 38.93 | 1431 | 202302172 |
| 8 | GS | Gansu | Zhangye | 100.45 | 38.93 | 1431 | 202302173 |
| 9 | GS | Gansu | Zhangye | 100.45 | 38.93 | 1431 | 202302174 |
| 10 | GS | Gansu | Zhangye | 100.45 | 38.93 | 1431 | 202302175 |
| 11 | GS | Gansu | Gaotai | 99.85 | 39.38 | 1402 | 202303221 |
| 12 | GS | Gansu | Gaotai | 99.85 | 39.38 | 1402 | 202303222 |
| 13 | GS | Gansu | Gaotai | 99.85 | 39.38 | 1402 | 202303223 |
| 14 | GS | Gansu | Gaotai | 99.85 | 39.38 | 1402 | 202303224 |
| 15 | GS | Gansu | Gaotai | 99.85 | 39.38 | 1402 | 202303225 |
| 16 | GS | Gansu | Jiuquan | 98.49 | 39.73 | 1489 | 202304291 |
| 17 | GS | Gansu | Jiuquan | 98.49 | 39.73 | 1489 | 202304292 |
| 18 | GS | Gansu | Jiuquan | 98.49 | 39.73 | 1489 | 202304293 |
| 19 | GS | Gansu | Jiuquan | 98.49 | 39.73 | 1489 | 202304294 |
| 20 | GS | Gansu | Jiuquan | 98.49 | 39.73 | 1489 | 202304295 |
| 21 | HB | Hebei | Shijiazhuang | 114.51 | 38.04 | 80 | 2302031 |
| 22 | HB | Hebei | Shijiazhuang | 114.51 | 38.04 | 80 | 2302032 |
| 23 | HB | Hebei | Shijiazhuang | 114.51 | 38.04 | 80 | 2302033 |
| 24 | HB | Hebei | Shijiazhuang | 114.51 | 38.04 | 80 | 2302034 |
| 25 | HB | Hebei | Shijiazhuang | 114.51 | 38.04 | 80 | 2302035 |
| 26 | HB | Hebei | Baoding | 115.49 | 38.89 | 44 | 2303011 |
| 27 | HB | Hebei | Baoding | 115.49 | 38.89 | 44 | 2303012 |
| 28 | HB | Hebei | Baoding | 115.49 | 38.89 | 44 | 2303013 |
| 29 | HB | Hebei | Baoding | 115.49 | 38.89 | 44 | 2303014 |
| 30 | HB | Hebei | Baoding | 115.49 | 38.89 | 44 | 2303015 |
| 31 | HB | Hebei | Xingtai | 114.50 | 37.07 | 35 | 2301071 |
| 32 | HB | Hebei | Xingtai | 114.50 | 37.07 | 35 | 2301072 |
| 33 | HB | Hebei | Xingtai | 114.50 | 37.07 | 35 | 2301073 |
| 34 | HB | Hebei | Xingtai | 114.50 | 37.07 | 35 | 2301074 |
| 35 | HB | Hebei | Xingtai | 114.50 | 37.07 | 35 | 2301075 |
| 36 | HB | Hebei | Huanghua | 117.33 | 38.37 | 3 | 2304081 |
| 37 | HB | Hebei | Huanghua | 117.33 | 38.37 | 3 | 2304082 |
| 38 | HB | Hebei | Huanghua | 117.33 | 38.37 | 3 | 2304083 |
| 39 | HB | Hebei | Huanghua | 117.33 | 38.37 | 3 | 2304084 |
| 40 | HB | Hebei | Huanghua | 117.33 | 38.37 | 3 | 2304085 |
| 41 | SD | Shandong | Taian | 117.13 | 36.19 | 153 | 230201 |
| 42 | SD | Shandong | Taian | 117.13 | 36.19 | 153 | 230202 |
| 43 | SD | Shandong | Taian | 117.13 | 36.19 | 153 | 230203 |
| 44 | SD | Shandong | Taian | 117.13 | 36.19 | 153 | 230204 |
| 45 | SD | Shandong | Taian | 117.13 | 36.19 | 153 | 230205 |
| 46 | SD | Shandong | Laiwu | 117.68 | 36.21 | 61 | 230101 |
| 47 | SD | Shandong | Laiwu | 117.68 | 36.21 | 61 | 230102 |
| 48 | SD | Shandong | Laiwu | 117.68 | 36.21 | 61 | 230103 |
| 49 | SD | Shandong | Laiwu | 117.68 | 36.21 | 61 | 230104 |
| 50 | SD | Shandong | Laiwu | 117.68 | 36.21 | 61 | 230105 |
| 51 | SD | Shandong | Binzhou | 117.97 | 37.38 | 10 | 230301 |
| 52 | SD | Shandong | Binzhou | 117.97 | 37.38 | 10 | 230302 |
| 53 | SD | Shandong | Binzhou | 117.97 | 37.38 | 10 | 230303 |
| 54 | SD | Shandong | Binzhou | 117.97 | 37.38 | 10 | 230304 |
| 55 | SD | Shandong | Binzhou | 117.97 | 37.38 | 10 | 230305 |
| 56 | SD | Shandong | Leling | 117.23 | 37.73 | 41 | 230601 |
| 57 | SD | Shandong | Leling | 117.23 | 37.73 | 41 | 230602 |
| 58 | SD | Shandong | Leling | 117.23 | 37.73 | 41 | 230603 |
| 59 | SD | Shandong | Leling | 117.23 | 37.73 | 41 | 230604 |
| 60 | SD | Shandong | Leling | 117.23 | 37.73 | 41 | 230605 |
| 61 | HN | Henan | Xinzheng | 113.72 | 34.39 | 113 | 2023121 |
| 62 | HN | Henan | Xinzheng | 113.72 | 34.39 | 113 | 2023122 |
| 63 | HN | Henan | Xinzheng | 113.72 | 34.39 | 113 | 2023123 |
| 64 | HN | Henan | Xinzheng | 113.72 | 34.39 | 113 | 2023124 |
| 65 | HN | Henan | Xinzheng | 113.72 | 34.39 | 113 | 2023125 |
| 66 | HN | Henan | Xinzheng | 113.72 | 34.39 | 113 | 2023126 |
| 67 | HN | Henan | Xinzheng | 113.72 | 34.39 | 113 | 2023127 |
| 68 | HN | Henan | Lingbao | 110.89 | 34.52 | 379 | 2326281 |
| 69 | HN | Henan | Lingbao | 110.89 | 34.52 | 379 | 2326282 |
| 70 | HN | Henan | Lingbao | 110.89 | 34.52 | 379 | 2326283 |
| 71 | HN | Henan | Lingbao | 110.89 | 34.52 | 379 | 2326284 |
| 72 | HN | Henan | Lingbao | 110.89 | 34.52 | 379 | 2326285 |
| 73 | HN | Henan | Lingbao | 110.89 | 34.52 | 379 | 2326286 |
| 74 | HN | Henan | Lingbao | 110.89 | 34.52 | 379 | 2326287 |
| 75 | HN | Henan | Zhoukou | 114.70 | 33.63 | 49 | 2326288 |
| 76 | HN | Henan | Zhoukou | 114.70 | 33.63 | 49 | 2326289 |
| 77 | HN | Henan | Zhoukou | 114.70 | 33.63 | 49 | 2326290 |
| 78 | HN | Henan | Zhoukou | 114.70 | 33.63 | 49 | 2326291 |
| 79 | HN | Henan | Zhoukou | 114.70 | 33.63 | 49 | 2326292 |
| 80 | HN | Henan | Zhoukou | 114.70 | 33.63 | 49 | 2326293 |
| 81 | SN | Shanxi | Yulin | 109.74 | 38.29 | 1043 | 2307071 |
| 82 | SN | Shanxi | Yulin | 109.74 | 38.29 | 1043 | 2307072 |
| 83 | SN | Shanxi | Yulin | 109.74 | 38.29 | 1043 | 2307073 |
| 84 | SN | Shanxi | Yulin | 109.74 | 38.29 | 1043 | 2307074 |
| 85 | SN | Shanxi | Yulin | 109.74 | 38.29 | 1043 | 2307075 |
| 86 | SN | Shanxi | Yulin | 109.74 | 38.29 | 1043 | 2307076 |
| 87 | SN | Shanxi | Yanan | 109.49 | 36.59 | 1074 | 2305221 |
| 88 | SN | Shanxi | Yanan | 109.49 | 36.59 | 1074 | 2305222 |
| 89 | SN | Shanxi | Yanan | 109.49 | 36.59 | 1074 | 2305223 |
| 90 | SN | Shanxi | Yanan | 109.49 | 36.59 | 1074 | 2305224 |
| 91 | SN | Shanxi | Yanan | 109.49 | 36.59 | 1074 | 2305225 |
| 92 | SN | Shanxi | Yanan | 109.49 | 36.59 | 1074 | 2305226 |
| 93 | SN | Shanxi | Yanan | 109.49 | 36.59 | 1074 | 2305227 |
| 94 | SN | Shanxi | Weinan | 109.51 | 34.50 | 403 | 2307081 |
| 95 | SN | Shanxi | Weinan | 109.51 | 34.50 | 403 | 2307082 |
| 96 | SN | Shanxi | Weinan | 109.51 | 34.50 | 403 | 2307083 |
| 97 | SN | Shanxi | Weinan | 109.51 | 34.50 | 403 | 2307084 |
| 98 | SN | Shanxi | Weinan | 109.51 | 34.50 | 403 | 2307085 |
| 99 | SN | Shanxi | Weinan | 109.51 | 34.50 | 403 | 2307086 |
| 100 | SN | Shanxi | Weinan | 109.51 | 34.50 | 403 | 2307087 |
| 101 | XJ | Xinjiang | Akesu | 80.26 | 41.17 | 1115 | 232131 |
| 102 | XJ | Xinjiang | Akesu | 80.26 | 41.17 | 1115 | 232132 |
| 103 | XJ | Xinjiang | Akesu | 80.26 | 41.17 | 1115 | 232133 |
| 104 | XJ | Xinjiang | Akesu | 80.26 | 41.17 | 1115 | 232134 |
| 105 | XJ | Xinjiang | Akesu | 80.26 | 41.17 | 1115 | 232135 |
| 106 | XJ | Xinjiang | Akesu | 80.26 | 41.17 | 1115 | 232136 |
| 107 | XJ | Xinjiang | Akesu | 80.26 | 41.17 | 1115 | 232137 |
| 108 | XJ | Xinjiang | Bayinguoleng | 88.17 | 39.02 | 1257 | 233211 |
| 109 | XJ | Xinjiang | Bayinguoleng | 88.17 | 39.02 | 1257 | 233212 |
| 110 | XJ | Xinjiang | Bayinguoleng | 88.17 | 39.02 | 1257 | 233213 |
| 111 | XJ | Xinjiang | Bayinguoleng | 88.17 | 39.02 | 1257 | 233214 |
| 112 | XJ | Xinjiang | Bayinguoleng | 88.17 | 39.02 | 1257 | 233215 |
| 113 | XJ | Xinjiang | Bayinguoleng | 88.17 | 39.02 | 1257 | 233216 |
| 114 | XJ | Xinjiang | Bayinguoleng | 88.17 | 39.02 | 1257 | 233217 |
| 115 | XJ | Xinjiang | Hetian | 79.93 | 37.11 | 1190 | 234181 |
| 116 | XJ | Xinjiang | Hetian | 79.93 | 37.11 | 1190 | 234182 |
| 117 | XJ | Xinjiang | Hetian | 79.93 | 37.11 | 1190 | 234183 |
| 118 | XJ | Xinjiang | Hetian | 79.93 | 37.11 | 1190 | 234184 |
| 119 | XJ | Xinjiang | Hetian | 79.93 | 37.11 | 1190 | 234185 |
| 120 | XJ | Xinjiang | Hetian | 79.93 | 37.11 | 1190 | 234186 |

Note: GS, Gansu; HB, Hebei; SD, Shandong; SN, Shaanxi; HN, Henan; XJ, Xinjiang.

Table. S2 The specific steps of the the KPCA dimension reduction algorithm.

clc;

clear all;

close all;

%% 1. Load data

% Note: The data samples are arranged by rows (samples) and features (parameters) are columns.

% The feature dimension should match the sample dimension.

tztqxk = xlsread('KPCA1.xlsx');

[n, p] = size(tztqxk); % n rows (samples), p columns (features)

data = tztqxk'; % If samples are rows, transpose so samples are columns

%% 2. Data normalization

X0 = zscore(data); % Standardize the data (mean 0, variance 1)

%% 3. Compute the kernel matrix

% Radial Basis Function (RBF) kernel

c = 2.5; % RBF parameter

% The choice of the RBF parameter can be optimized (e.g., with genetic algorithms or PSO)

for i = 1:p

for j = 1:p

K(i, j) = exp(-(norm(X0(i, :) - X0(j, :)))^2 / (2 * c^2));

K(j, i) = K(i, j);

end

end

%% 4. Center the matrix

unit = (1/p) * ones(p, p);

Kp = K - unit*K - K*unit + unit*K*unit;

%% 5. Eigen decomposition

% Eigenvectors (proper_vector) and eigenvalues (proper_value)

[proper_vector, proper_value] = eig(Kp);

% Extract the diagonal elements of the eigenvalue matrix

proper_value_vec = diag(proper_value);

proper_value_vec = abs(proper_value_vec);

% Sort eigenvalues in descending order

[proper_value_sort, index] = sort(proper_value_vec, 'descend');

%% 6. Contribution rate calculation

contribution_rate = proper_value_sort * 100 / sum(proper_value_sort); % Proportion of total variance explained

cum_contribution_rate = (cumsum(proper_value_sort) / sum(proper_value_sort)) * 100; % Cumulative contribution rate

com = 0;

for rem = 1:length(contribution_rate)

com = com + contribution_rate(rem);

if com > 90

break;

end

end

oo = proper_vector(:, index); % Eigenvectors ordered by descending eigenvalues

%% 6.1 Eigenvectors with respect to sorted eigenvalues (transposed and rotated 90 degrees)

V = rot90(proper_vector)';

disp(proper_vector);

%% 6.2 Optional normalization of eigenvectors

% V = zscore(V);

%% 7. Plotting

% 7.1 Plot eigenvalues

figure('color', [1 1 1]);

plot(proper_value_sort, 'b.-', 'MarkerSize', 10, 'linewidth', 1);

ylim = get(gca, 'Ylim'); % Get current y-axis range

hold on

x = 1:p;

y = ones(1, p);

plot(x, y, 'r--', 'linewidth', 1);

box off

set(gca, 'linewidth', 1)

set(gca, 'FontSize', 10); % Font size for axis labels, legends, titles, etc.

set(get(gca, 'Xlabel'), 'FontSize', 10);

set(get(gca, 'Ylabel'), 'FontSize', 10);

set(gca, 'XColor', 'k', 'YColor', 'k');

legend('Eigenvalues', 'Eigenvalue = 1');

xlabel('Number of principal components', 'FontWeight', 'bold');

ylabel('Eigenvalue', 'FontWeight', 'bold');

print(gcf, '-r600', '-djpeg', 'KPCA_Eigenvalue_curve.jpg'); % Save as JPEG

% 7.2 Plot cumulative contribution rate

figure('color', [1 1 1]);

[~, ~] = hist(contribution_rate); % Note: this line computes a histogram but its output is unused

bar(contribution_rate, 'linewidth', 1);

box off

set(gca, 'linewidth', 1)

set(gca, 'FontSize', 10);

set(get(gca, 'Xlabel'), 'FontSize', 10);

set(get(gca, 'Ylabel'), 'FontSize', 10);

set(gca, 'XColor', 'k', 'YColor', 'k');

xlabel('Number of principal components', 'FontWeight', 'bold');

ylabel('Contribution rate (%)', 'FontWeight', 'bold');

for i = 1:rem

text(i, contribution_rate(i) + 2, num2str(contribution_rate(i)), 'VerticalAlignment', 'middle', 'HorizontalAlignment', 'center');

end

print(gcf, '-r600', '-djpeg', 'KPCA_Contribution_Rate.jpg'); % Save as JPEG

%% 8. Compute the principal component values we need

a = rem; % Here, we keep enough principal components to exceed 90% cumulative contribution

% a is the number of principal components to retain

F = zeros(n, a); % Initialize matrix to store principal components (each column is a principal component)

% F is n×a

% n is the number of samples, a is the number of principal components

% Note: a principal component is a weighted sum of eigenvectors and eigenvalues

% Ai is n×a

% Z is also n×a, so we need to transpose

for i = 1:a

ai = oo(:, i)'; % Take the i-th sorted eigenvector and transpose to a row vector

Ai = repmat(ai, n, 1); % Repeat this row vector n times to form an n×p matrix

F(:, i) = sum(Ai .* X0', 2); % Compute weighted sum for each sample using the standardized data

end

proper_value_data = [proper_value, contribution_rate, cum_contribution_rate];

xlswrite('KPCA_x_Principal_Component_vectors.xlsx', oo); % Save the ordered eigenvectors

xlswrite('KPCA_x_Principal_Component_Data.xlsx', F);

xlswrite('KPCA_x_Principal_Component_Contributions.xlsx', proper_value_data);

**Table. S3** Metabolic component Information of CJ samples by LC-MS/MS.

| ID | Name | MZ | RT | Exact_mass | ppm | Formula | Pos/Neg |
| --- | --- | --- | --- | --- | --- | --- | --- |
| M387T223 | 1-O-Sinapoyl-beta-D-glucose | 387.1274 | 222.7 | 386.1213 | 3.03775 | C_17_H_22_O_10_ | pos |
| M369T265_1 | 4-O-beta-D-Glucosyl-sinapate | 369.1164 | 264.8 | 386.1213 | 6.86509 | C_17_H_22_O_10_ | pos |
| M335T465 | Prostaglandin A2 | 335.2234 | 464.9 | 334.2144 | 5.142866 | C_20_H_30_O_4_ | pos |
| M172T233 | Iminoarginine | 172.0969 | 233.2 | 172.096 | 5.229639 | C_6_H_12_N_4_O_2_ | pos |
| M295T460 | 13(S)-HOT | 295.2261 | 460.1 | 294.2195 | 2.289765 | C_18_H_30_O_3_ | pos |
| M275T357 | Etherolenic acid | 275.2008 | 356.8 | 292.2038 | 16.11213 | C_18_H_28_O_3_ | pos |
| M313T422 | 9,12,13-TriHOME | 313.2367 | 421.6 | 330.2406 | 11.28233 | C_18_H_34_O_5_ | pos |
| M313T388 | Octadec-9-ene-1,18-dioic-acid | 313.2362 | 387.7 | 312.2301 | 3.754341 | C_18_H_32_O_4_ | pos |
| M355T436 | Bufadienolide | 355.2625 | 435.7 | 354.2559 | 1.902815 | C_24_H_34_O_2_ | pos |
| M329T364 | 17beta-Acetamidoandrost-4-en-3-one | 329.2305 | 364.3 | 329.2355 | 15.1867 | C_21_H_31_NO_2_ | pos |
| M102T489 | Triethylamine | 102.127 | 489.4 | 101.1204 | 5.890935 | C_6_H_15_N | pos |
| M102T422 | 2-Ketobutyric acid | 102.0338 | 422.4 | 102.0317 | 3.748122 | C_4_H_6_O_3_ | pos |
| M104T51_1 | Dimethylglycine | 104.0705 | 51.4 | 103.0633 | 0.730274 | C_4_H_9_NO_2_ | pos |
| M109T418 | m-Cresol | 109.1008 | 417.7 | 108.0575 | 6.079863 | C_7_H_8_O | pos |
| M110T39 | Hydroquinone | 110.02 | 38.6 | 110.0368 | 1.389267 | C_6_H_6_O_2_ | pos |
| M110T381 | Catechol | 110.0214 | 380.8 | 110.0368 | 9.163832 | C_6_H_6_O_2_ | pos |
| M131T251 | Ketoleucine | 130.9652 | 251.3 | 130.063 | 8.464796 | C_6_H_1_0O_3_ | pos |
| M131T36 | Heptanoic acid | 130.9658 | 36.3 | 130.0994 | 1.669724 | C_7_H_1_4O_2_ | pos |
| M114T301 | Creatinine | 113.9621 | 301.1 | 113.0589 | 16.6449 | C_4_H_7_N_3_O | pos |
| M116T84 | L-Proline | 116.0686 | 83.6 | 115.0633 | 17.02441 | C_5_H_9_NO_2_ | pos |
| M124T556 | Picolinic acid | 124.0861 | 556.5 | 123.032 | 0.710349 | C_6_H_5_NO_2_ | pos |
| M124T686 | 3-Hydroxybenzyl alcohol glucoside | 124.0859 | 686.2 | 124.0524 | 9.057497 | C_7_H_8_O_2_ | pos |
| M126T412 | Ciliatine | 125.9865 | 412.1 | 125.0242 | 0.210255 | C_2_H_8_NO_3_P | pos |
| M126T336 | Thymine | 125.9861 | 335.9 | 126.0429 | 4.899145 | C_5_H_6_N_2_O_2_ | pos |
| M146T58_1 | 4-Guanidinobutanoic acid | 146.0923 | 57.6 | 145.0851 | 0.520219 | C_5_H_11_N_3_O_2_ | pos |
| M130T78 | Pipecolic acid | 130.0498 | 77.9 | 129.079 | 18.73681 | C_6_H_11_NO_2_ | pos |
| M132T505 | (R)-Pantolactone | 131.5336 | 505.2 | 130.063 | 0.001114 | C_6_H_10_O_3_ | pos |
| M131T146 | Creatine | 130.9665 | 145.6 | 131.0695 | 1.218219 | C_4_H_9_N_3_O_2_ | pos |
| M132T176 | Glutaric acid | 131.9751 | 176.3 | 132.0423 | 6.954881 | C_5_H_8_O_4_ | pos |
| M132T63 | L-Leucine | 132.1018 | 63.2 | 131.0946 | 0.575314 | C_6_H_13_NO_2_ | pos |
| M138T219 | Tyramine | 138.0912 | 218.6 | 137.0841 | 0.012906 | C_8_H_11_NO | pos |
| M142T111 | O-Phosphoethanolamine | 141.9585 | 110.7 | 141.0191 | 1.262335 | C_2_H_8_NO_4_P | pos |
| M145T682 | Anabasine | 144.9826 | 681.9 | 162.1157 | 0.597122 | C_10_H_14_N_2_ | pos |
| M147T681 | 4-Hydroxycinnamic acid | 146.9815 | 681.4 | 164.0473 | 10.13062 | C_9_H_8_O_3_ | pos |
| M147T393 | L-Lysine | 147.1168 | 392.7 | 146.1055 | 23.91807 | C_6_H_14_N_2_O_2_ | pos |
| M149T36 | L-2-Hydroxyglutaric acid | 148.9759 | 36 | 148.0372 | 7.622189 | C_5_H_8_O_5_ | pos |
| M149T235 | L-Methionine | 148.9754 | 235.4 | 149.051 | 13.1294 | C_5_H_11_NO_2_S | pos |
| M152T265 | Pyridoxine | 152.0705 | 264.8 | 169.0739 | 26.52717 | C_8_H_11_NO_3_ | pos |
| M155T422 | 2,3-Butanediol | 154.9925 | 421.9 | 154.0122 | 13.76689 | C_4_H_10_O_2_S_2_ | pos |
| M156T47 | L-Histidine | 156.0767 | 47 | 155.0695 | 0.48694 | C_6_H_9_N_3_O_2_ | pos |
| M175T47 | L-Arginine | 175.1187 | 47 | 174.1117 | 1.433733 | C_6_H_14_N_4_O_2_ | pos |
| M159T181 | 4,5-Dihydroorotic acid | 158.9589 | 180.7 | 158.0328 | 14.54564 | C_5_H_6_N_2_O_4_ | pos |
| M159T135 | Allantoin | 158.9616 | 135.4 | 158.044 | 12.53466 | C_4_H_6_N_4_O_3_ | pos |
| M145T245 | 3-Hydroxymethylglutaric acid | 145.0487 | 244.7 | 162.0528 | 22.98538 | C_6_H_10_O_5_ | pos |
| M163T33 | Acetylcysteine | 162.9977 | 32.8 | 163.0303 | 1.206149 | C_5_H_9_NO_3_S | pos |
| M166T303 | L-Phenylalanine | 166.0861 | 302.8 | 165.079 | 1.059691 | C_9_H_11_NO_2_ | pos |
| M183T277 | 4-Pyridoxic acid | 182.9821 | 277.2 | 183.0532 | 17.80691 | C_8_H_9_NO_4_ | pos |
| M166T152 | 3-Methylxanthine | 166.0495 | 152.4 | 166.0491 | 2.40892 | C_6_H_6_N_4_O_2_ | pos |
| M167T44 | L-4-Hydroxyphenylglycine | 167.0119 | 44.1 | 167.0582 | 5.854679 | C_8_H_9_NO_3_ | pos |
| M167T383 | Quinolinic acid | 167.0132 | 382.9 | 167.0219 | 0.674046 | C_7_H_5_NO_4_ | pos |
| M170T145 | Norepinephrine | 170.0811 | 145.5 | 169.0739 | 0.446846 | C_8_H_11_NO_3_ | pos |
| M187T382 | Undecanoic acid | 186.9524 | 382 | 186.162 | 20.95332 | C_11_H_22_O_2_ | pos |
| M171T281 | 1-Hydroxy-2-naphthoate | 171.1488 | 280.8 | 188.0473 | 0.467458 | C_11_H_8_O_3_ | pos |
| M188T312 | N-Alpha-acetyllysine | 188.0705 | 312.2 | 188.1161 | 1.242327 | C_8_H_16_N_2_O_3_ | pos |
| M172T36 | 3-Dehydroshikimate | 171.9945 | 36 | 172.0372 | 7.013999 | C_7_H_8_O_5_ | pos |
| M177T220 | N-Formyl-L-methionine | 176.972 | 220.1 | 177.046 | 0.00069 | C_6_H_11_NO_3_S | pos |
| M195T223 | trans-Ferulic acid | 195.0653 | 223.3 | 194.0579 | 0.009606 | C_10_H_10_O_4_ | pos |
| M181T34 | Acetylcholine chloride | 180.9889 | 33.6 | 181.087 | 5.77084 | C_7_H_16_NO_2_. C_l_ | pos |
| M182T78 | L-Tyrosine | 182.0808 | 77.8 | 181.0739 | 2.065017 | C_9_H_11_NO_3_ | pos |
| M183T177 | Sorbitol | 182.9851 | 177.3 | 182.079 | 1.116734 | C_6_H_14_O_6_ | pos |
| M183T348 | Mannitol | 182.9878 | 348.5 | 182.079 | 3.040354 | C_6_H_14_O_6_ | pos |
| M200T33 | Dodecanoic acid | 199.9751 | 32.6 | 200.1776 | 0.01172 | C_12_H_24_O_2_ | pos |
| M208T303 | N-Acetyl-L-phenylalanine | 208.0964 | 303.1 | 207.0895 | 1.806855 | C_11_H_13_NO_3_ | pos |
| M209T463 | L-Kynurenine | 209.1535 | 463.2 | 208.0848 | 0.841598 | C_10_H_12_N_2_O_3_ | pos |
| M218T321 | N-Acetylserotonin | 218.2115 | 320.7 | 218.1055 | 1.696764 | C_12_H_14_N_2_O_2_ | pos |
| M224T380 | Hydroxykynurenine | 223.984 | 380.2 | 224.0797 | 19.72059 | C_10_H_12_N_2_O_4_ | pos |
| M228T652 | Deoxyuridine | 228.1967 | 652.5 | 228.0746 | 3.205007 | C_9_H_12_N_2_O_5_ | pos |
| M243T180 | 1-Hexadecanol | 243.1822 | 179.7 | 242.261 | 0.648847 | C_16_H_34_O | pos |
| M226T653 | Porphobilinogen | 226.18 | 652.9 | 226.0954 | 2.126435 | C_10_H_14_N_2_O_4_ | pos |
| M248T254 | Pyridoxal 5'-phosphate | 248.1283 | 253.8 | 247.0246 | 17.81718 | C_8_H_10_NO_6_P | pos |
| M257T288 | (2S)-Liquiritigenin | 257.1855 | 288.2 | 256.0736 | 18.21855 | C_15_H_12_O_4_ | pos |
| M281T502 | Linoleic acid | 281.2469 | 502.4 | 280.2402 | 2.048023 | C_18_H_32_O_2_ | pos |
| M271T532 | Genistein | 271.2263 | 531.6 | 270.0528 | 3.47486 | C_15_H_10_O_5_ | pos |
| M291T330 | Androsterone | 291.1975 | 330.2 | 290.2246 | 4.263275 | C_19_H_30_O_2_ | pos |
| M282T580 | (9E)-Octadecenoic acid | 282.2787 | 580.3 | 282.2559 | 1.754467 | C_18_H_34_O_2_ | pos |
| M284T78_2 | Guanosine | 284.0973 | 78 | 283.0917 | 5.899387 | C_10_H_13_N_5_O_5_ | pos |
| M300T249 | all-trans-Retinoic acid | 300.1983 | 249.4 | 300.2089 | 1.233313 | C_20_H_28_O_2_ | pos |
| M290T407 | Epiandrosterone | 290.2677 | 407 | 290.2246 | 4.264604 | C_19_H_30_O_2_ | pos |
| M303T355 | Quercetin | 303.0493 | 355.4 | 302.043 | 3.220598 | C_15_H_10_O_7_ | pos |
| M312T643 | Arachidic acid | 312.3257 | 643.3 | 312.3028 | 1.587258 | C_20_H_40_O_2_ | pos |
| M336T601 | 12-Keto-tetrahydro-leukotriene B4 | 336.3127 | 601.5 | 336.2301 | 8.279573 | C_20_H_32_O_4_ | pos |
| M321T611 | Pregnanediol | 321.3136 | 611.2 | 320.2715 | 4.596383 | C_21_H_36_O_2_ | pos |
| M343T413_1 | Sucrose | 343.2941 | 413.4 | 342.1162 | 1.928703 | C_12_H_22_O_11_ | pos |
| M355T620 | Prostaglandin F2a | 355.2804 | 619.8 | 354.2406 | 5.213367 | C_20_H_34_O_5_ | pos |
| M346T78_1 | GMP | 346.0536 | 78 | 363.058 | 8.767428 | C_10_H_14_N_5_O_8_P | pos |
| M377T260 | Riboflavin | 377.1445 | 259.5 | 376.1383 | 1.922723 | C_17_H_20_N_4_O_6_ | pos |
| M385T601 | Vitamin D3 | 385.2923 | 601.2 | 384.3392 | 1.828588 | C_27_H_44_O | pos |
| M408T652 | Allocholic acid | 408.3649 | 651.6 | 408.2876 | 5.59067 | C_24_H_40_O_5_ | pos |
| M415T474_2 | Sodium deoxycholate | 415.211 | 473.6 | 414.2746 | 1.496902 | C_24_H_39_O_4_. Na | pos |
| M430T303 | alpha-Tocopherol | 430.2417 | 303.3 | 430.3811 | 2.526068 | C_29_H_50_O_2_ | pos |
| M545T650 | Protoporphyrin IX | 545.3962 | 649.6 | 562.258 | 8.463863 | C_34_H_34_N_4_O_4_ | pos |
| M122T257 | N,N-Dimethylaniline | 122.0961 | 257.4 | 121.0891 | 2.260514 | C_8_H_11_N | pos |
| M123T78 | Niacinamide | 123.0554 | 77.7 | 122.048 | 0.812642 | C_6_H_6_N_2_O | pos |
| M127T188 | Maltol | 127.0388 | 188.2 | 126.0317 | 1.385404 | C_6_H_6_O_3_ | pos |
| M136T56 | Adenine | 136.0616 | 56.3 | 135.0545 | 1.293532 | C_5_H_5_N_5_ | pos |
| M138T241 | p-Aminobenzoic acid | 138.0549 | 241.5 | 137.0477 | 0.550506 | C_7_H_7_NO_2_ | pos |
| M143T192 | Kojic acid | 143.033 | 192.4 | 142.0266 | 6.124461 | C_6_H_6_O_4_ | pos |
| M143T79 | trans-trans-Muconic acid | 143.0338 | 78.6 | 142.0266 | 0.531343 | C_6_H_6_O_4_ | pos |
| M174T288 | N-Acetylleucine | 174.1125 | 287.8 | 173.1052 | 0 | C_8_H_15_NO_3_ | pos |
| M197T278 | N-Acetylhistidine | 197.0808 | 278 | 197.08 | 4.059249 | C_8_H_11_N_3_O_3_ | pos |
| M180T84 | Metanephrine | 180.1015 | 83.9 | 197.1052 | 20.73275 | C_10_H_15_NO_3_ | pos |
| M207T305 | Sinapic acid | 207.0651 | 305.4 | 224.0685 | 19.4818 | C_11_H_12_O_5_ | pos |
| M255T516 | 16-Hydroxy hexadecanoic acid | 255.2315 | 516.1 | 272.2351 | 15.02166 | C_16_H_32_O_3_ | pos |
| M277T543 | Stearidonic acid | 277.2151 | 542.6 | 276.2089 | 3.881462 | C_18_H_28_O_2_ | pos |
| M279T663 | 13S-hydroxyoctadecadienoic acid | 279.2297 | 663.2 | 296.2351 | 7.284325 | C_18_H_32_O_3_ | pos |
| M282T657 | Oleamide | 282.2803 | 656.8 | 281.2719 | 3.981858 | C_18_H_35_NO | pos |
| M282T79_2 | 2'-O-Methyladenosine | 282.1181 | 78.9 |  | 5.671384 | C_11_H_16_N_5_O_7_P(C_5_H_8_O_6_PR)n(C_5_H_8_O_6_PR)n | pos |
| M284T678 | Octadecanamide | 284.2938 | 678.4 | 283.2875 | 3.433068 | C_18_H_37_NO | pos |
| M313T465 | 9(S)-HPODE | 313.2378 | 464.8 | 312.2301 | 1.353604 | C_18_H_32_O_4_ | pos |
| M295T613 | 13-L-Hydroperoxylinoleic acid | 295.2254 | 613.5 | 312.2301 | 6.435761 | C_18_H_32_O_4_ | pos |
| M300T599 | Palmitoylethanolamide | 300.2881 | 598.8 | 299.2824 | 5.248293 | C_18_H_37_NO_2_ | pos |
| M317T542 | Prostaglandin B2 | 317.2081 | 542 | 334.2144 | 3.57494 | C_20_H_30_O_4_ | pos |
| M337T475 | Prostaglandin E1 | 337.2386 | 474.9 | 354.2406 | 2.075682 | C_20_H_34_O_5_ | pos |
| M369T369 | Bufalin | 369.2292 | 368.9 | 386.2457 | 24.55385 | C_24_H_34_O_4_ | pos |
| M425T688 | Betulin | 425.3754 | 687.6 | 442.3811 | 4.076399 | C_30_H_50_O_2_ | pos |
| M461T615 | Galactosylsphingosine | 461.3223 | 614.6 | 461.3353 | 28.17986 | C_24_H_47_NO_7_ | pos |
| M495T617_1 | Mibefradil | 495.2978 | 617.1 | 495.2897 | 8.283818 | C_29_H_38_FN_3_O_3_ | pos |
| M507T648_2 | Retinyl palmitate | 507.4471 | 648.2 | 524.4593 | 9.392112 | C_36_H_60_O_2_ | pos |
| M130T60 | Pyroglutamic acid | 130.0498 | 60 | 129.0426 | 0.584392 | C_5_H_7_NO_3_ | pos |
| M137T78 | Hypoxanthine | 137.0456 | 77.7 | 136.0385 | 1.284244 | C_5_H_4_N_4_O | pos |
| M146T83 | 4-Acetamidobutanoic acid | 146.0813 | 83.1 | 145.0739 | 0.848842 | C_6_H_11_NO_3_ | pos |
| M206T89 | Xanthurenic acid | 206.0448 | 89.5 | 205.0375 | 0.11648 | C_10_H_7_NO_4_ | pos |
| M647T600_2 | Uridine diphosphate glucuronic acid | 647.4618 | 600.3 | 580.0343 | 8.967861 | C_15_H_22_N_2_O_18_P_2_ | pos |
| M102T459 | Ethylmethylacetic acid | 102.127 | 458.7 | 102.0681 | 8.206789 | C_5_H_10_O_2_ | pos |
| M432T373 | Chenodeoxycholic acid glycine conjugate | 432.3072 | 373.2 | 449.3141 | 1.235233 | C_26_H_43_NO_5_ | pos |
| M133T62 | L-Asparagine | 133.0607 | 62.2 | 132.0535 | 0.511917 | C_4_H_8_N_2_O_3_ | pos |
| M300T446 | Phytosphingosine | 300.2888 | 446 | 317.293 | 10.76963 | C_18_H_39_NO_3_ | pos |
| M471T447_2 | Asiatic acid | 471.3537 | 447 | 488.3502 | 23.19702 | C_30_H_48_O_5_ | pos |
| M100T112 | N-Methyl-2-pyrrolidinone | 100.0756 | 111.8 | 99.0684 | 0.759426 | C_5_H_9_NO | pos |
| M138T55 | N-Methylnicotinate | 138.0549 | 55 | 137.0477 | 0.550506 | C_7_H_7_NO_2_ | pos |
| M193T310 | Scopoletin | 193.0495 | 309.9 | 192.0423 | 0.393681 | C_10_H_8_O_4_ | pos |
| M180T76 | 3-Succinoylpyridine | 180.0651 | 75.9 | 179.0582 | 2.088134 | C_9_H_9_NO_3_ | pos |
| M183T482 | Benzophenone | 183.0804 | 482.3 | 182.0732 | 0.415118 | C_13_H_10_O | pos |
| M185T277 | Sebacic acid | 185.117 | 276.6 | 202.1205 | 9.496673 | C_10_H_18_O_4_ | pos |
| M208T82 | Pilocarpine | 208.1199 | 81.8 | 208.1212 | 6.246399 | C_11_H_16_N_2_O_2_ | pos |
| M213T277 | Harmine | 213.1016 | 276.7 | 212.095 | 2.005798 | C_13_H_12_N_2_O | pos |
| M251T669 | OPEO | 251.1993 | 668.7 | 250.1933 | 5.000332 | C_16_H_26_O_2_ | pos |
| M261T554 | (9Z,12Z,15Z)-Octadecatrienoic acid | 261.2204 | 553.8 | 278.2246 | 12.38035 | C_18_H_30_O_2_ | pos |
| M280T443 | Stearolic acid | 280.2344 | 442.6 | 280.2402 | 20.69696 | C_18_H_32_O_2_ | pos |
| M279T525 | (9Z,11E,13E)-Octadecatrienoic acid | 279.2306 | 525 | 278.2246 | 4.5697 | C_18_H_30_O_2_ | pos |
| M285T371 | 9-cis-Retinal | 285.2065 | 371.5 | 284.214 | 0.655237 | C_20_H_28_O | pos |
| M297T475 | 12,13-DHOME | 297.2437 | 474.9 | 314.2457 | 18.2813 | C_18_H_34_O_4_ | pos |
| M298T675 | Tridemorph | 298.31 | 675.5 | 297.3032 | 1.595656 | C_19_H_39_NO | pos |
| M323T409 | Clostebol | 323.1826 | 408.9 | 322.17 | 16.60416 | C_19_H_27_ClO_2_ | pos |
| M326T611 | Oleoylethanolamide | 326.3052 | 610.6 | 325.2981 | 0.539372 | C_20_H_39_NO_2_ | pos |
| M311T309 | N-Desmethylcitalopram | 311.1477 | 308.7 | 310.1481 | 24.66996 | C_19_H_19_FN_2_O | pos |
| M439T629_2 | Oleanolic acid | 439.3564 | 628.8 | 456.3603 | 8.043584 | C_30_H_48_O_3_ | pos |
| M453T448_1 | Glycyrrhetinate | 453.3342 | 448 | 470.3396 | 4.486756 | C_30_H_46_O_4_ | pos |
| M117T76 | Glutarate semialdehyde | 117.0547 | 75.9 | 116.0473 | 1.059334 | C_5_H_8_O_3_ | pos |
| M119T201 | 5-Hydroxypentanoic acid | 119.0729 | 200.6 | 118.063 | 22.03692 | C_5_H_10_O_3_ | pos |
| M127T57 | Triacetate lactone | 127.0389 | 57.3 | 126.0317 | 0.598242 | C_6_H_6_O_3_ | pos |
| M128T294_2 | D-1-Piperideine-2-carboxylic acid | 128.0705 | 294.3 | 127.0633 | 0.593423 | C_6_H_9_NO_2_ | pos |
| M140T492 | Acetylphosphate | 139.9874 | 491.7 | 139.9875 | 0.71435 | C_2_H_5_O_5_P | pos |
| M161T298 | N(6)-Methyllysine | 161.1324 | 297.7 | 160.1212 | 0.199755 | C_7_H_16_N_2_O_2_ | pos |
| M145T57 | 2-Keto-glutaramic acid | 145.0495 | 56.7 | 145.0375 | 0.109221 | C_5_H_7_NO_4_ | pos |
| M156T126 | DHHA | 156.0654 | 126.2 | 155.0582 | 0.486975 | C_7_H_9_NO_3_ | pos |
| M160T312 | Indoleacetaldehyde | 160.0756 | 312.3 | 159.0684 | 0.011788 | C_10_H_9_NO | pos |
| M160T219 | 4-Acetamido-2-aminobutanoic acid | 160.0937 | 219.3 | 160.0848 | 19.08871 | C_6_H_12_N_2_O_3_ | pos |
| M165T241 | m-Coumaric acid | 165.0547 | 241.2 | 164.0473 | 0.009332 | C_9_H_8_O_3_ | pos |
| M170T280 | 8-Amino-7-oxononanoate | 170.1173 | 280 | 187.1208 | 23.12522 | C_9_H_17_NO_3_ | pos |
| M172T90 | Tetrahydrodipicolinate | 172.0603 | 89.7 | 171.0532 | 0.141229 | C_7_H_9_NO_4_ | pos |
| M192T425 | N,N-Diethyl-m-toluamide | 192.1379 | 425 | 191.131 | 0.486884 | C_12_H_17_NO | pos |
| M179T278 | (Z)-4-Hydroxy-6-dodecenoic acid lactone | 179.0701 | 277.7 | 178.0477 | 0.249362 | C_6_H_10_O_6_ | pos |
| M179T218 | Coniferyl aldehyde | 179.0698 | 218 | 178.063 | 2.658181 | C_10_H_10_O_3_ | pos |
| M179T537 | (1S,2R,4S)-(-)-Bornyl acetate | 179.1429 | 536.5 | 196.1463 | 22.51834 | C_12_H_20_O_2_ | pos |
| M183T421_1 | Se-Methylselenocysteine | 182.9876 | 420.6 | 182.9799 | 11.60244 | C_4_H_9_NO_2_Se | pos |
| M183T228 | 5-Oxo-1,2-campholide | 183.1013 | 227.5 | 182.0943 | 1.507362 | C_10_H_14_O_3_ | pos |
| M188T78_2 | 2-Keto-6-acetamidocaproate | 188.0917 | 77.7 | 187.0845 | 0.404058 | C_8_H_13_NO_4_ | pos |
| M195T388 | Neocnidilide | 195.1379 | 388.3 | 194.1307 | 0.01343 | C_12_H_18_O_2_ | pos |
| M196T109 | 2-Amino-2-deoxy-D-gluconate | 196.0971 | 108.9 | 195.0743 | 0.654453 | C_6_H_13_NO_6_ | pos |
| M202T201_1 | Thiabendazole | 202.0439 | 200.6 | 201.0361 | 1.608148 | C_10_H_7_N_3_S | pos |
| M202T367 | Spermine | 202.2163 | 367.4 | 202.2157 | 2.96712 | C_10_H_26_N_4_ | pos |
| M204T81 | N-Acetyl-D-glucosamine | 204.0865 | 80.9 | 221.0899 | 19.76613 | C_8_H_15_NO_6_ | pos |
| M206T276 | Indolelactic acid | 206.0812 | 276.2 | 205.0739 | 0.002846 | C_11_H_11_NO_3_ | pos |
| M209T340 | trans-Isoasarone | 209.1172 | 340.1 | 208.1099 | 0.114768 | C_12_H_16_O_3_ | pos |
| M216T145 | Kinetin | 216.0866 | 144.7 | 215.0807 | 4.419208 | C_10_H_9_N_5_O | pos |
| M219T488 | Capsidiol | 219.174 | 488 | 236.1776 | 0.265966 | C_15_H_24_O_2_ | pos |
| M221T297_2 | Caryophyllene alpha-oxide | 221.1898 | 297.4 | 220.1827 | 0.002932 | C_15_H_24_O | pos |
| M223T514 | (2E,6E)-Farnesol | 223.2052 | 513.8 | 222.1984 | 1.070304 | C_15_H_26_O | pos |
| M227T267 | Biotin | 227.0811 | 266.7 | 244.0882 | 1.47084 | C_10_H_16_N_2_O_3_S | pos |
| M229T380 | Traumatic Acid | 229.1433 | 379.6 | 228.1362 | 0.022453 | C_12_H_20_O_4_ | pos |
| M232T92 | N-Acetyl-L-2-amino-6-oxopimelate | 232.0815 | 92.1 | 231.0743 | 0.327471 | C_9_H_13_NO_6_ | pos |
| M240T81 | Procarbazine hydrochloride | 240.1223 | 80.9 | 257.1295 | 0.974503 | C_12_H_19_N_3_O. HCl | pos |
| M247T356 | alpha-Santonin | 247.1322 | 356.2 | 246.1256 | 1.799253 | C_15_H_18_O_3_ | pos |
| M249T437 | Juvenile hormone III | 249.1853 | 436.8 | 266.1882 | 18.19529 | C_16_H_26_O_3_ | pos |
| M270T446 | 16-Oxopalmitate | 270.2149 | 446.1 | 270.2195 | 17.02349 | C_16_H_30_O_3_ | pos |
| M276T60 | 4-Hydroxycinnamoylagmatine | 276.1444 | 59.6 | 276.1586 | 1.230793 | C_14_H_20_N_4_O_2_ | pos |
| M261T60 | Glucose 6-phosphate | 261.0367 | 60.5 | 260.0297 | 0.048447 | C_6_H_13_O_9_P | pos |
| M265T299 | (S)-Abscisic acid | 265.1428 | 298.7 | 264.1362 | 0.014407 | C_15_H_20_O_4_ | pos |
| M267T499 | (10S)-Juvenile hormone III diol | 267.1955 | 499.5 | 284.1988 | 15.47182 | C_16_H_28_O_4_ | pos |
| M277T285 | Aspartame | 277.118 | 285.3 | 294.1216 | 13.83526 | C_14_H_18_N_2_O_5_ | pos |
| M293T446_2 | 13(S)-HpOTrE | 293.2117 | 446.3 | 310.2144 | 16.11311 | C_18_H_30_O_4_ | pos |
| M296T52_1 | Dehypoxanthine futalosine | 296.0981 | 52 | 296.0896 | 28.7067 | C_14_H_16_O_7_ | pos |
| M315T475 | 9,10-DHOME | 315.2535 | 474.6 | 314.2457 | 1.662154 | C_18_H_34_O_4_ | pos |
| M303T281 | Delphinidin | 303.0504 | 281.2 | 303.0505 | 0.329978 | C_15_H_11_O_7_ | pos |
| M309T365 | Bilobalide A | 309.0867 | 364.5 | 326.1002 | 19.62556 | C_15_H_18_O_8_ | pos |
| M309T446 | 2,3-Dinor-8-iso prostaglandin F2alpha | 309.204 | 446 | 326.2093 | 1.780582 | C_18_H_30_O_5_ | pos |
| M331T464 | Carnosol | 331.1866 | 464.2 | 330.1831 | 4.247537 | C_20_H_26_O_4_ | pos |
| M318T498 | Chlorpromazine | 318.0976 | 498.4 | 318.0957 | 5.97301 | C_17_H_19_ClN_2_S | pos |
| M327T330 | Pentaporphyrin I | 327.1603 | 329.5 | 327.1471 | 3.036235 | C_19_H_21_NO_4_ | pos |
| M335T528 | Prostaglandin-c2 | 335.2191 | 528.4 | 334.2144 | 0.154201 | C_20_H_30_O_4_ | pos |
| M336T342 | Berberine | 336.1214 | 342.3 | 336.1236 | 2.83546 | C_20_H_18_NO_4_ | pos |
| M351T497_2 | 11-Dehydro-thromboxane B2 | 351.2135 | 496.6 | 368.2199 | 2.944078 | C_20_H_32_O_6_ | pos |
| M353T418_1 | (13E)-11a-Hydroxy-9,15-dioxoprost-13-enoic acid | 353.2291 | 417.6 | 352.225 | 0.418898 | C_20_H_32_O_5_ | pos |
| M411T630 | 5-Dehydroavenasterol | 411.3617 | 630.5 | 410.3549 | 1.157133 | C_29_H_46_O | pos |
| M442T579 | 4a-Carboxy-4b-methyl-5a-cholesta-8,24-dien-3b-ol | 442.3558 | 578.6 | 442.3447 | 6.354583 | C_29_H_46_O_3_ | pos |
| M467T451_1 | Cholesterol sulfate | 467.3137 | 450.8 | 466.3117 | 11.29006 | C_27_H_46_O_4_S | pos |
| M455T498_2 | Ribostamycin | 455.2377 | 498.1 | 454.2275 | 6.423018 | C_17_H_34_N_4_O_10_ | pos |
| M537T660_3 | Alpha-Carotene | 537.3997 | 659.6 | 536.4382 | 27.60472 | C_40_H_56_ | pos |
| M553T617_2 | beta-Cryptoxanthin | 553.4435 | 616.9 | 552.4331 | 5.644659 | C_40_H_56_O | pos |
| M267T59 | 2-O-(alpha-D-Mannosyl)-D-glycerate | 267.0719 | 58.9 | 268.0794 | 0.838725 | C_9_H_16_O_9_ | neg |
| M283T395 | Xanthosine | 283.0672 | 395.2 | 284.0757 | 4.324043 | C_10_H_12_N_4_O_6_ | neg |
| M193T59 | D-Glucuronic acid | 193.0353 | 58.8 | 194.0427 | 0.642369 | C_6_H_10_O_7_ | neg |
| M221T59 | 6-Acetyl-D-glucose | 221.0664 | 59 | 222.074 | 1.465621 | C_8_H_14_O_7_ | neg |
| M178T88 | Galactonolactone | 178.0519 | 88.1 | 178.0477 | 23.58918 | C_6_H_10_O_6_ | neg |
| M177T82 | 2-Dehydro-3-deoxy-D-gluconate | 177.0406 | 82.4 | 178.0477 | 0.994123 | C_6_H_10_O_6_ | neg |
| M160T218 | 3D-3,5_4-Trihydroxycyclohexane-1,2-dione | 160.0404 | 218.3 | 160.0372 | 19.99535 | C_6_H_8_O_5_ | neg |
| M147T58 | L-Xylonate | 147.0299 | 58.2 | 166.0477 | 4.012805 | C_5_H_10_O_6_ | neg |
| M105T50 | Glyceric acid | 105.0198 | 50.1 | 106.0266 | 4.532479 | C_3_H_6_O_4_ | neg |
| M113T211 | Dihydrouracil | 112.9856 | 210.5 | 114.0429 | 10.09931 | C_4_H_6_N_2_O_2_ | neg |
| M113T479 | 2-Heptanone | 112.9857 | 479.4 | 114.1045 | 19.28998 | C_7_H_14_O | neg |
| M115T46 | Fumaric acid | 115.0038 | 46.5 | 116.011 | 0.660848 | C_4_H_4_O_4_ | neg |
| M117T689 | Betaine | 116.9272 | 689.2 | 117.079 | 0.371183 | C_5_H_11_NO_2_ | neg |
| M121T96 | Benzoate | 121.03 | 96.5 | 122.0368 | 3.932909 | C_7_H_6_O_2_ | neg |
| M122T108 | Nicotinic acid | 122.0252 | 107.9 | 123.032 | 3.900834 | C_6_H_5_NO_2_ | neg |
| M124T373 | 1-Naphthylamine | 123.9015 | 372.9 | 143.0735 | 3.842853 | C_10_H_9_N | neg |
| M124T90_2 | Taurine | 124.0406 | 90.3 | 125.0147 | 18.05095 | C_2_H_7_NO_3_S | neg |
| M133T46 | L-Malic acid | 133.0132 | 46.5 | 134.0215 | 0.909104 | C_4_H_6_O_5_ | neg |
| M135T383 | Phenylacetic acid | 134.8655 | 383.3 | 136.0524 | 13.17049 | C_8_H_8_O_2_ | neg |
| M135T665 | Phenyl acetate | 134.8952 | 665.2 | 136.0524 | 14.11994 | C_8_H_8_O_2_ | neg |
| M159T164 | Oxoadipic acid | 158.9789 | 164 | 160.0372 | 10.9653 | C_6_H_8_O_5_ | neg |
| M144T210 | Spermidine | 143.9168 | 209.5 | 145.1579 | 15.93689 | C_7_H_19_N_3_ | neg |
| M146T50 | N-Methyl-D-aspartic acid | 146.0459 | 50.2 | 147.0532 | 0.164332 | C_5_H_9_NO_4_ | neg |
| M147T473 | trans-Cinnamate | 146.9662 | 472.9 | 148.0524 | 6.944159 | C_9_H_8_O_2_ | neg |
| M150T677 | L-Arabinose | 149.9934 | 677.1 | 150.0528 | 1.369526 | C_5_H_10_O_5_ | neg |
| M150T641 | D-Lyxose | 149.9949 | 641.4 | 150.0528 | 11.36985 | C_5_H_10_O_5_ | neg |
| M150T267 | D-Ribose | 150.0563 | 266.7 | 150.0528 | 23.32458 | C_5_H_10_O_5_ | neg |
| M169T104 | 3,4-Dihydroxyphenylglycol | 169.0511 | 104.2 | 170.0579 | 2.815717 | C_8_H_10_O_4_ | neg |
| M152T95 | 3-Amino-4-hydroxybenzoate | 152.0354 | 95.3 | 153.0426 | 0.499884 | C_7_H_7_NO_3_ | neg |
| M153T247 | Gentisic acid | 153.0182 | 247.4 | 154.0266 | 1.702824 | C_7_H_6_O_4_ | neg |
| M173T59_1 | Shikimic acid | 173.0445 | 58.8 | 174.0528 | 0.910818 | C_7_H_10_O_5_ | neg |
| M174T47 | N-Acetyl-L-aspartic acid | 174.0408 | 46.6 | 175.0481 | 0.137899 | C_6_H_9_NO_5_ | neg |
| M161T114 | D-Mannose | 161.0418 | 113.6 | 180.0634 | 19.93271 | C_6_H_12_O_6_ | neg |
| M188T58 | Azelaic acid | 188.0926 | 58.5 | 188.1049 | 15.19988 | C_9_H_16_O_4_ | neg |
| M172T210_1 | 4-Quinolinecarboxylic acid | 171.9091 | 209.9 | 173.0477 | 0.847403 | C_10_H_7_NO_2_ | neg |
| M191T78_1 | Citric acid | 191.0195 | 78.2 | 192.027 | 1.172655 | C_6_H_8_O_7_ | neg |
| M175T74 | Guanidinosuccinic acid | 174.9562 | 74 | 175.0593 | 7.284468 | C_5_H_9_N_3_O_4_ | neg |
| M175T141 | Ascorbate | 175.0249 | 141.2 | 176.0321 | 0.434224 | C_6_H_8_O_6_ | neg |
| M177T59 | Gluconolactone | 177.0405 | 58.8 | 178.0477 | 0 | C_6_H_10_O_6_ | neg |
| M179T55 | D-Fructose | 179.0568 | 54.6 | 180.0634 | 3.775338 | C_6_H_12_O_6_ | neg |
| M188T117 | Kynurenic acid | 188.0357 | 116.7 | 189.0426 | 1.99962 | C_10_H_7_NO_3_ | neg |
| M195T62 | Gluconic acid | 195.0506 | 62.3 | 196.0583 | 0.295353 | C_6_H_12_O_7_ | neg |
| M223T441 | Methyl jasmonate | 223.1338 | 441.3 | 224.1412 | 0.55572 | C_13_H_20_O_3_ | neg |
| M223T434 | Thymidine | 223.029 | 434 | 242.0903 | 4.966247 | C_10_H_14_N_2_O_5_ | neg |
| M243T59_2 | Uridine | 243.061 | 58.8 | 244.0695 | 3.848519 | C_9_H_12_N_2_O_6_ | neg |
| M227T600 | Myristic acid | 227.2013 | 600.2 | 228.2089 | 0.168323 | C_14_H_28_O_2_ | neg |
| M255T676 | Palmitic acid | 255.2337 | 675.8 | 256.2402 | 3.040351 | C_16_H_32_O_2_ | neg |
| M267T510 | Formononetin | 267.1971 | 510.4 | 268.0736 | 20.62389 | C_16_H_12_O_4_ | neg |
| M269T541 | Dehydroepiandrosterone | 269.2121 | 541.4 | 288.2089 | 0.394225 | C_19_H_28_O_2_ | neg |
| M281T664 | (6Z)-Octadecenoic acid | 281.2477 | 664.4 | 282.2559 | 0.10184 | C_18_H_34_O_2_ | neg |
| M297T564 | Nonadecanoic acid | 297.2429 | 564.3 | 298.2872 | 2.108917 | C_19_H_38_O_2_ | neg |
| M309T686 | Ribose 1,5-bisphosphate | 309.1689 | 686.3 | 309.9855 | 16.927 | C_5_H_12_O_11_P_2_ | neg |
| M342T78 | Melibiose | 342.1197 | 78.2 | 342.1162 | 10.23034 | C_12_H_22_O_11_ | neg |
| M341T55_2 | Trehalose | 341.1078 | 54.7 | 342.1162 | 2.426836 | C_12_H_22_O_11_ | neg |
| M339T677 | Fructose 1,6-bisphosphate | 339.1975 | 676.9 | 339.996 | 6.597387 | C_6_H_14_O_12_P_2_ | neg |
| M365T526 | Nervonic acid | 365.2305 | 525.9 | 366.3498 | 1.000679 | C_24_H_46_O_2_ | neg |
| M471T585_2 | Maslinic acid | 471.3526 | 585.3 | 472.3553 | 5.809479 | C_30_H_48_O_4_ | neg |
| M119T53 | L-Erythrulose | 119.035 | 53.1 | 120.0423 | 0 | C_4_H_8_O_4_ | neg |
| M101T603 | Succinic acid semialdehyde | 101.0236 | 602.7 | 102.0317 | 7.918942 | C_4_H_6_O_3_ | neg |
| M129T45 | Itaconic acid | 129.0192 | 45.2 | 130.0266 | 0.775078 | C_5_H_6_O_4_ | neg |
| M131T78_1 | L-Ribulose | 131.0336 | 77.9 | 150.0528 | 6.181621 | C_5_H_10_O_5_ | neg |
| M135T62 | Threonic acid | 135.0298 | 62.1 | 136.0372 | 0.740577 | C_4_H_8_O_5_ | neg |
| M137T163 | Salicylic acid | 137.0245 | 162.6 | 138.0317 | 0.554645 | C_7_H_6_O_3_ | neg |
| M149T52 | D-Xylose | 149.0457 | 52 | 150.0528 | 1.180846 | C_5_H_10_O_5_ | neg |
| M151T78 | Xanthine | 151.026 | 78.2 | 152.0334 | 0.662138 | C_5_H_4_N_4_O_2_ | neg |
| M179T244 | Alpha-D-Glucose | 179.0516 | 243.6 | 180.0634 | 25.13242 | C_6_H_12_O_6_ | neg |
| M161T130 | Fructose-1P | 161.0419 | 129.7 | 180.0634 | 19.31174 | C_6_H_12_O_6_ | neg |
| M161T279 | L-Gulose | 161.0418 | 279.1 | 180.0634 | 19.93271 | C_6_H_12_O_6_ | neg |
| M167T165 | 3,4-Dihydroxybenzeneacetic acid | 167.0353 | 165.5 | 168.0423 | 1.652345 | C_8_H_8_O_4_ | neg |
| M191T63 | Glucaric acid | 191.02 | 63.4 | 210.0376 | 4.135693 | C_6_H_10_O_8_ | neg |
| M229T287 | Dodecanedioic acid | 229.1446 | 287.5 | 230.1518 | 0.331668 | C_12_H_22_O_4_ | neg |
| M265T610 | Practolol | 265.148 | 610.2 | 266.163 | 29.1309 | C_14_H_22_N_2_O_3_ | neg |
| M293T527_1 | 9-OxoODE | 293.2109 | 526.7 | 294.2195 | 4.497855 | C_18_H_30_O_3_ | neg |
| M279T567 | Bovinic acid | 279.2371 | 567.5 | 280.2402 | 14.68286 | C_18_H_32_O_2_ | neg |
| M328T485_1 | Labetalol | 328.1827 | 485 | 328.1787 | 12.18833 | C_19_H_24_N_2_O_3_ | neg |
| M310T562 | 11Z-Eicosenoic acid | 310.2821 | 561.6 | 310.2872 | 16.43666 | C_20_H_38_O_2_ | neg |
| M325T543 | 11-Dehydrocorticosterone | 325.1861 | 543.1 | 344.1988 | 17.49767 | C_21_H_28_O_4_ | neg |
| M328T79_1 | Cyclic AMP | 328.0435 | 78.8 | 329.0525 | 5.18224 | C_10_H_12_N_5_O_6_P | neg |
| M339T574 | Behenic acid | 339.3256 | 574 | 340.3341 | 3.607155 | C_22_H_44_O_2_ | neg |
| M409T490 | LysoPA(16_0_0_0) | 409.2354 | 490.3 | 410.2433 | 1.524795 | C_19_H_39_O_7_P | neg |
| M457T211_1 | Amygdalin | 457.1537 | 210.8 | 457.1584 | 10.28101 | C_20_H_27_NO_11_ | neg |
| M503T56 | 1-Kestose | 503.1593 | 56.1 | 504.169 | 4.81756 | C_18_H_32_O_16_ | neg |
| M198T486 | Procollagen 5-hydroxy-L-lysine | 197.8083 | 485.9 |  | 1.29199 | C_7_H_13_N_3_O_3_R_2_ | neg |
| M151T345 | 3-Hydroxyphenylacetic acid | 151.0398 | 344.7 | 152.0473 | 1.483053 | C_8_H_8_O_3_ | neg |
| M161T231 | D-Glucose | 161.0454 | 230.6 | 180.0634 | 2.421677 | C_6_H_12_O_6_ | neg |
| M171T472 | Capric acid | 171.139 | 472.1 | 172.1463 | 0.140237 | C_10_H_20_O_2_ | neg |
| M253T624 | Palmitoleic acid | 253.2165 | 624.3 | 254.2246 | 3.175938 | C_16_H_30_O_2_ | neg |
| M281T515 | Vaccenic acid | 281.2521 | 515 | 282.2559 | 12.35902 | C_18_H_34_O_2_ | neg |
| M283T568 | Stearic acid | 283.2645 | 567.7 | 284.2715 | 0.867062 | C_18_H_36_O_2_ | neg |
| M367T635_2 | Tetracosanoic acid | 367.3577 | 635 | 368.3654 | 1.154188 | C_24_H_48_O_2_ | neg |
| M369T505 | Quassin | 369.1599 | 504.6 | 388.1886 | 27.92828 | C_22_H_28_O_6_ | neg |
| M455T599 | Ursolic acid | 455.3565 | 599.1 | 456.3603 | 7.533886 | C_30_H_48_O_3_ | neg |
| M125T124 | 1,2,3-Trihydroxybenzene | 125.0246 | 124.4 | 126.0317 | 1.407723 | C_6_H_6_O_3_ | neg |
| M137T242 | Gentisate aldehyde | 137.0251 | 242.2 | 138.0317 | 4.933403 | C_7_H_6_O_3_ | neg |
| M165T128 | Phenyllactate | 165.0558 | 127.9 | 166.063 | 0.46045 | C_9_H_10_O_3_ | neg |
| M161T260 | beta-D-Fructose | 161.0418 | 260.2 | 180.0634 | 19.93271 | C_6_H_12_O_6_ | neg |
| M165T244 | Terephthalate | 165.0182 | 243.8 | 166.0266 | 6.81137 | C_8_H_6_O_4_ | neg |
| M173T62 | N-Methyltryptamine | 173.1045 | 62.5 | 174.1157 | 22.66839 | C_11_H_14_N_2_ | neg |
| M285T331 | Hexadecanedioate | 285.2077 | 331.1 | 286.2144 | 2.019581 | C_16_H_30_O_4_ | neg |
| M289T239 | Catechin | 289.0706 | 239.1 | 290.079 | 3.888323 | C_15_H_14_O_6_ | neg |
| M298T532 | Ostruthin | 298.1554 | 531.8 | 298.1569 | 5.012733 | C_19_H_22_O_3_ | neg |
| M385T265 | Gardenoside | 385.1139 | 264.9 | 404.1319 | 1.012687 | C_17_H_24_O_11_ | neg |
